# Cognitive Deficits Associated with Na_v_1.1 Alterations: Involvement of Neuronal Firing Dynamics and Oscillations

**DOI:** 10.1371/journal.pone.0151538

**Published:** 2016-03-15

**Authors:** Alex C. Bender, Bryan W. Luikart, Pierre-Pascal Lenck-Santini

**Affiliations:** 1 Department of Neurology, Geisel School of Medicine at Dartmouth, Lebanon, NH, United States of America; 2 Department of Physiology & Neurobiology, Geisel School of Medicine at Dartmouth, Lebanon, NH, United States of America; 3 Department of Neurological Sciences, University of Vermont, Burlington, VT, United States of America; 4 Institut de Neurobiologie de la Méditerranée, INSERM, Marseille, France; Georgia State University, UNITED STATES

## Abstract

Brain oscillations play a critical role in information processing and may, therefore, be essential to uncovering the mechanisms of cognitive impairment in neurological disease. In Dravet syndrome (DS), a mutation in *SCN1A*, coding for the voltage-gated sodium channel Na_v_1.1, is associated with severe cognitive impairment and seizures. While seizure frequency and severity do not correlate with the extent of impairment, the slowing of brain rhythms may be involved. Here we investigate the role of Na_v_1.1 on brain rhythms and cognition using RNA interference. We demonstrate that knockdown of Na_v_1.1 impairs fast- and burst-firing properties of neurons in the medial septum *in vivo*. The proportion of neurons that fired phase-locked to hippocampal theta oscillations was reduced, and medial septal regulation of theta rhythm was disrupted. During a working memory task, this deficit was characterized by a decrease in theta frequency and was negatively correlated with performance. These findings suggest a fundamental role for Na_v_1.1 in facilitating fast-firing properties in neurons, highlight the importance of precise temporal control of theta frequency for working memory, and imply that Na_v_1.1 deficits may disrupt information processing in DS via a dysregulation of brain rhythms.

## Introduction

There is now accumulating evidence that oscillatory activity in the brain plays an important role in cognitive function. Brain oscillations reflect the coordinated activity of collections of neurons. These oscillations represent, and may themselves influence, important temporal patterns in the brain. For example, in the rodent hippocampus, spatial information is organized by theta (5–12 Hz) oscillations with specific sequences of neurons, representing the path of the animal, being activated in each theta cycle [1,2]. In humans, theta oscillations are also correlated with spatial working memory, navigation, sensorimotor integration, and learning and recall [3–6]. As a result, theta oscillations have been postulated to provide a temporal structure for information processing [2,7,8]. These brain rhythms have an essential role in normal cognition, and an alteration of oscillations may lead to cognitive and behavioral impairment [9–13].

Here we investigate the possibility that an alteration of brain oscillatory activity may contribute to cognitive impairment in Dravet syndrome (DS). This lifelong epilepsy disorder is caused in the majority of cases by mutations in the gene *SCN1A*, coding for the voltage-gated sodium channel Na_v_1.1, and is characterized by both seizures and severe cognitive impairment [14–22]. While seizure frequency and severity do not correlate with the extent of impairment, evidence from human and animal studies suggests that a slowing of brain rhythms may be involved [23–26]. We hypothesized that oscillatory activity may be affected directly by Na_v_1.1 deficits and that cognitive impairment may arise, at least in part, by altered oscillations.

To test this hypothesis, we used a RNA-interference (RNAi)-mediated approach to selectively target the septo-hippocampal network in the intact, adult rat brain, thereby avoiding neuropathological and developmental abnormalities observed with genetic deletion of Na_v_1.1 (e.g. seizures and motor impairment) [27–29]. The septo-hippocampal network is comprised of the medial septum and diagonal band of Broca (MSDB) and the hippocampal formation, which have reciprocal connections to each other via the fimbria fornix [30]. In particular, neurons in the MSDB are essential for the regulation of hippocampal theta oscillations [31–33], and therefore this network as a whole plays a central role in learning and memory processes [34–37].

We specifically tested the effects of MSDB Na_v_1.1 knockdown on neuronal firing properties, hippocampal theta rhythm, and spatial working memory performance in a T-maze alternation task. We found that MSDB knockdown of Na_v_1.1 was sufficient to induce working memory deficits, and that performance was correlated with the frequency of theta oscillations at the choice point of the maze. *In vivo* single-unit recordings performed in rats under urethane anesthesia revealed a specific alteration of fast-spiking and theta phase-locking properties of MSDB neurons. Our results suggest that reduction of Na_v_1.1 expression in the MSDB induces working memory impairment via a deficit of fast spiking neurons and subsequent alteration of rhythmic activity in septo-hippocampal networks. We propose that alterations of brain rhythms may contribute to cognitive impairment in Dravet syndrome.

## Materials and Methods

A detailed description of Materials and Methods can be found in S1 Methods.

### Animals

Male, adult (postnatal day 60–180), Sprague-Dawley rats (Charles River Laboratories, Wilmington, MA) were used. Animals were housed in standard facilities under USDA- and AAALAC-approved conditions with a 12:12 hour light:dark cycle and ad libitum access to food and water (except when indicated otherwise for behavioral procedures). Animals were anesthetized with isoflurane (1–3% in oxygen) for surgery or with intraperitoneal (i.p.) injection of 1.5 g/kg Urethane for single-unit recordings. After experiments, animals were euthanized by applying deep anesthesia with isoflurane followed by intracardial perfusion of saline and 4% PFA. All procedures were approved by the Dartmouth College Institutional Animal Care and Use Committee and were performed in accordance with the Institute for Laboratory Animal Research (ILAR) Guide for the Care and Use of Laboratory Animals.

### Generation of Lentiviral Vectors & Viral Injections

Two shRNA sequences targeting the rat *Scn1a* gene were used (sh-1: 5’ - CCAGAGCGATTATGTGACAAGCATT - 3’; sh-2: 5’ - AAAGAGAAACTCAACGAAA - 3’). The target sequence for sh-1 was previously validated as an siRNA [25]. Each shRNA expression sequence was cloned into the FUGW lentiviral vector and was driven by a U6 promoter. The FUGW vector also contained a downstream fluorescent reporter (Green Fluorescent Protein; GFP), and has been published previously [38]. The same vector containing the GFP expression sequence but no shRNA sequence was used as control. The lentivirus was packaged by calcium phosphate-mediated transfection of HEK293 FT cells. A total of 2 μl of the viral solution was infused into the MSDB from each hemisphere as described in S1 Methods, using the following target coordinates: AP +0.7, ML 0.0, DV 6.6 (mm from bregma).

### Quantitative Real-Time PCR & Immunohistochemistry

Analysis of *Scn1a* knockdown was performed in B50 neuroblastoma cells (HPA Cultures #85042302). Cells plated at equal density were harvested 4 days after infection with the lentivirus. RNA was extracted and used for real-time quantitative PCR (RT-PCR) with primer sets for *Scn1a* (Applied Biosystems Assay ID# Rn00578439), *Scn2a* (Rn00561862) and *GAPDH* (Rn99999916). *Scn1a* and *Scn2a* expression levels are reported as normalized to *GAPDH*.

Immunofluorescence was performed for analysis of Na_v_1.1 expression in the MSDB. Rats were anesthetized with isoflurane and perfused intracardially with phosphate buffered saline (PBS) followed by 4% paraformaldehyde (PFA). Immunofluorescence was performed on free-floating sections incubated with the following primary antibodies: rabbit anti-Na_v_1.1 (1:50, Chemicon/Millipore), mouse anti-parvalbumin (1:1000, Chemicon/Millipore), chicken anti-GFP (1:1000, Abcam), mouse anti-GAD67 (1:200, Chemicon/Millipre), or goat anti-choline acetyl-tranferase (ChAT; 1:500, Chemicon/Millipore).

### Single-Unit and LFP Recordings under Urethane

Between 2–6 weeks after MSDB injections, each rat was anesthetized by intraperitoneal (i.p.) injection of 1.5 g/kg Urethane (Sigma-Aldrich Co.) and placed in a stereotaxic frame. An LFP electrode was stereotaxically lowered into the dorsal hippocampus CA1 region (AP -4.0, ML 3.0(R), DV 2.8), and a 16-channel silicon probe (NeuroNexus Technologies) was lowered along the midline into the MSDB (AP +0.7). All electrodes were coated with DiI prior to implantation to facilitate visualization of the electrode tracks.

MSDB single-units and hippocampal LFPs were simultaneously recorded during 10 minute sessions, with the depth of the single-unit probe being lowered each time to span the vertical length of the MSDB. During each recording session, theta oscillations were typically observed spontaneously and were induced by pinching the tail of the rat, on average 3 times per session. Recordings for which the injection site was not located within the MSDB or the electrode track did not pass through the injection site were excluded.

### T-maze Rewarded Alternation & Hippocampal LFP Recordings

Rats that had been previously injected with the lentivirus containing either the control vector or sh*Scn1a*-2 (sh-2) were subsequently implanted with hippocampal LFP recording electrodes and were tested on a rewarded alternation (non-match-to-place) protocol in a T-maze.

On each test trial, rats were given a Sample run (a forced turn either left or right), followed by a variable delay period, and then a Choice run. Correct choices required the rat to choose the opposite arm on the Choice run. During the first 2 days of testing, there was no delay interval between the Sample and Choice runs, but on days 3 to 6, a variable delay was incorporated (15, 30 or 60 seconds. During behavioral testing, the rat’s position was tracked by two LEDs that were attached to the rat headstage, and hippocampal LFPs were recorded.

### Data Analysis

Single-unit discrimination was performed offline, and all analyses for single-units and LFP data were performed using custom-written programs in Matlab (Mathworks, Inc.). Statistical analyses are indicated in the text and in S1 Methods.

The proportion of units that exhibited fast-firing properties (including tonic- and burst-firing) were quantified by setting filters based on the peak and mean firing frequencies (see S1 Methods). For LFP data recorded under urethane anesthesia, time-frequency power spectra were computed using the multi-taper method (window size 4s, step 1s). To compare between rats, power values were normalized to the sum of the power spectrum. Theta epochs were extracted with an automatic theta detection algorithm based on the times when the normalized theta power exceeded an empirically-determined threshold of 0.94 a.u. For analysis of tail pinch events, 30s of LFP data were extracted time-locked to the tail pinch. For theta phase-locking of MSDB units, spikes that occurred outside of theta epochs were discarded. The mean resultant vector length was calculated from the theta phase of the spikes (circular statistics toolbox, Matlab), and units with significant theta phase-locking were identified using the Rayleigh test for non-uniformity [39].

For LFP data recorded during performance in the T-maze, time-frequency power spectra were computed using the short-time Fourier transform (window size 0.5s, step 0.1s). Theta frequency and normalized power were first evaluated as a function of binned running speed. To investigate the dynamic changes in theta rhythm, the maze was divided into 10 position bins and theta values were determined for each bin. Only periods of movement were included by applying a speed threshold of greater than 5 cm/s.

## Results

### Na_v_1.1 Expression in the MSDB

**Fig 1:**
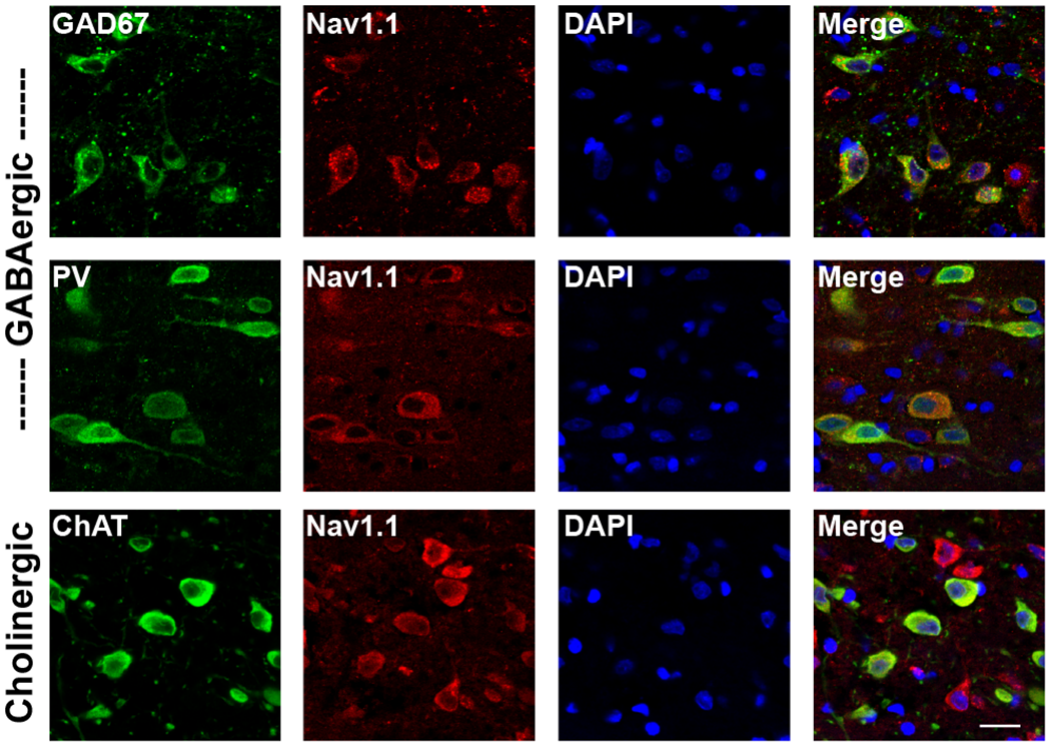
Na_v_1.1 is expressed in cholinergic and GABAergic neurons in the MSDB. Immunofluorescence for Na_v_1.1 and cell-type specific markers in the MSDB of an adult rat brain. Co-localization was observed with GAD67, PV and ChAT, indicating expression in both GABAergic and cholinergic neurons. Scale bar, 20 μm.

Immunohistochemical labeling indicated strong expression of Na_v_1.1 throughout the MSDB (S1A Fig), consistent with the high *in situ* hybridization signal shown in the Allen Brain Atlas (www.brain-map.org). Na_v_1.1 co-localized with cell-type specific markers for MSDB neurons, including glutamic acid decarboxylase-67 (GAD67), parvalbumin (PV) and choline acetyl-transferase (ChAT; Fig 1 and S1B Fig). Quantification revealed that the majority (89%) of cholinergic neurons (120/134 counted), and nearly all (99%) GABAergic neurons (152/154 counted), including those expressing PV (199/202 counted, 99%), expressed Na_v_1.1. Although Na_v_1.1 has been shown to be expressed predominantly in GABAergic neurons in cortical regions [27,40], recent evidence indicates that some forebrain excitatory neurons also express Na_v_1.1 [41], as do serotonergic and cholinergic neurons in sub-cortical regions [42], consistent with our observations in the MSDB.

### shRNA-mediated Knockdown of Na_v_1.1

**Fig 2:**
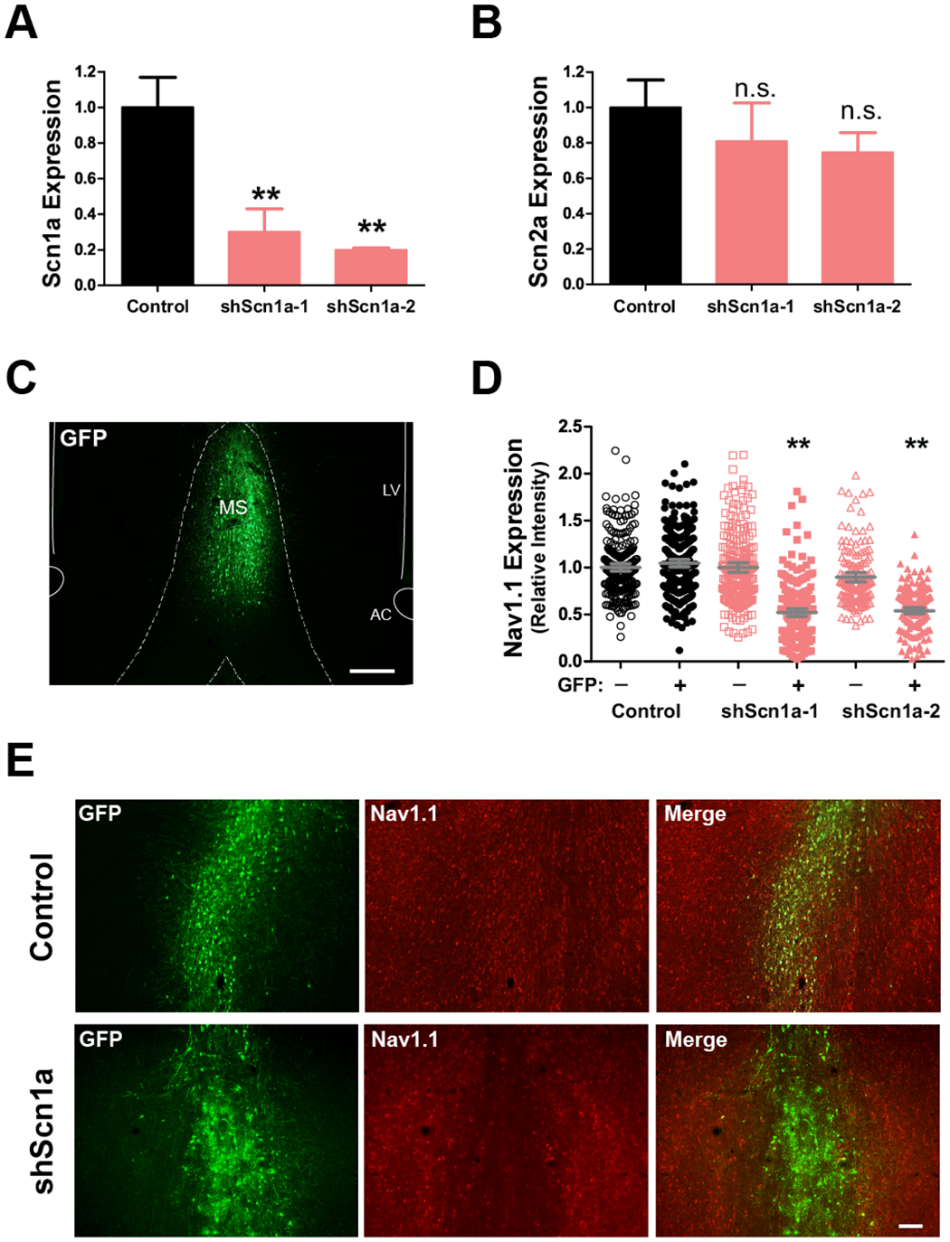
shRNA-mediated knockdown of Na_v_1.1. Lentiviral vectors were generated for two separate shRNAs targeting the rat *Scn1a* gene (sh-1 and sh-2). (A-B) Lentiviral vectors were tested in B50 neuroblastoma cells for efficient knockdown of the target gene. (A) Both shRNAs reduced *Scn1a* expression by approximately 70–80% compared to a control vector, (B) but did not affect expression of a related sodium channel gene, *Scn2a*. Mean +/- SEM, **p<.01. (C) Example of the MSDB injection site showing expression of the GFP reporter sequence in infected cells. Scale bar, 200 μm. (D) Quantification of mean fluorescent intensity per cell for Na_v_1.1 signal in GFP-positive and GFP-negative MSDB cells. Grey bars show mean +/- SEM, **p<.01. (E) Example of Na_v_1.1 immunofluorescence in the MSDB of a control and shScn1a injected rat showing a reduction in Na_v_1.1 fluorescent signal in neurons infected with the shScn1a, but not control, vector. Scale bar, 100 μm.

Two lentiviral vectors were generated. Each encoded a different shRNA sequence targeted to the *Scn1a* gene (sh-1 and sh-2) and driven by the U6 promoter. Each vector also contained a downstream fluorescent reporter (GFP) driven by the ubiquitin promoter. Both shRNAs achieved a 70–80% reduction in *Scn1a* expression in B50 neuroblastoma cells *in vitro* compared to a control vector expressing only GFP (F(2,6) = 12.65, p<.01; Fig 2A). Expression of a related sodium channel gene, *Scn2a*, was not affected (F(2,6) = 0.56, p>.05; Fig 2B). Injection of the lentivirus into the MSDB of adult rats resulted in substantial infectivity of the target region, as indicated by GFP expression, and little to no infection of surrounding regions (Fig 2C). Immunofluorescence also revealed a reduction in Na_v_1.1 labeling in the infected region of the MSDB compared to controls expressing only GFP (Fig 2D and 2E). Quantitative analysis of GFP-positive cells showed a reduction in Na_v_1.1 fluorescent signal of approximately 50% for both shRNAs compared to GFP-negative cells in the same sections or compared to control animals infected with lentivirus expressing GFP-only (F(5,1323) = 125.9, n = 1329 cells from 3 rats per group, p<.01; Fig 2D).

### Reduction of Fast-spiking Discharge in MSDB neurons *In Vivo*

**Fig 3:**
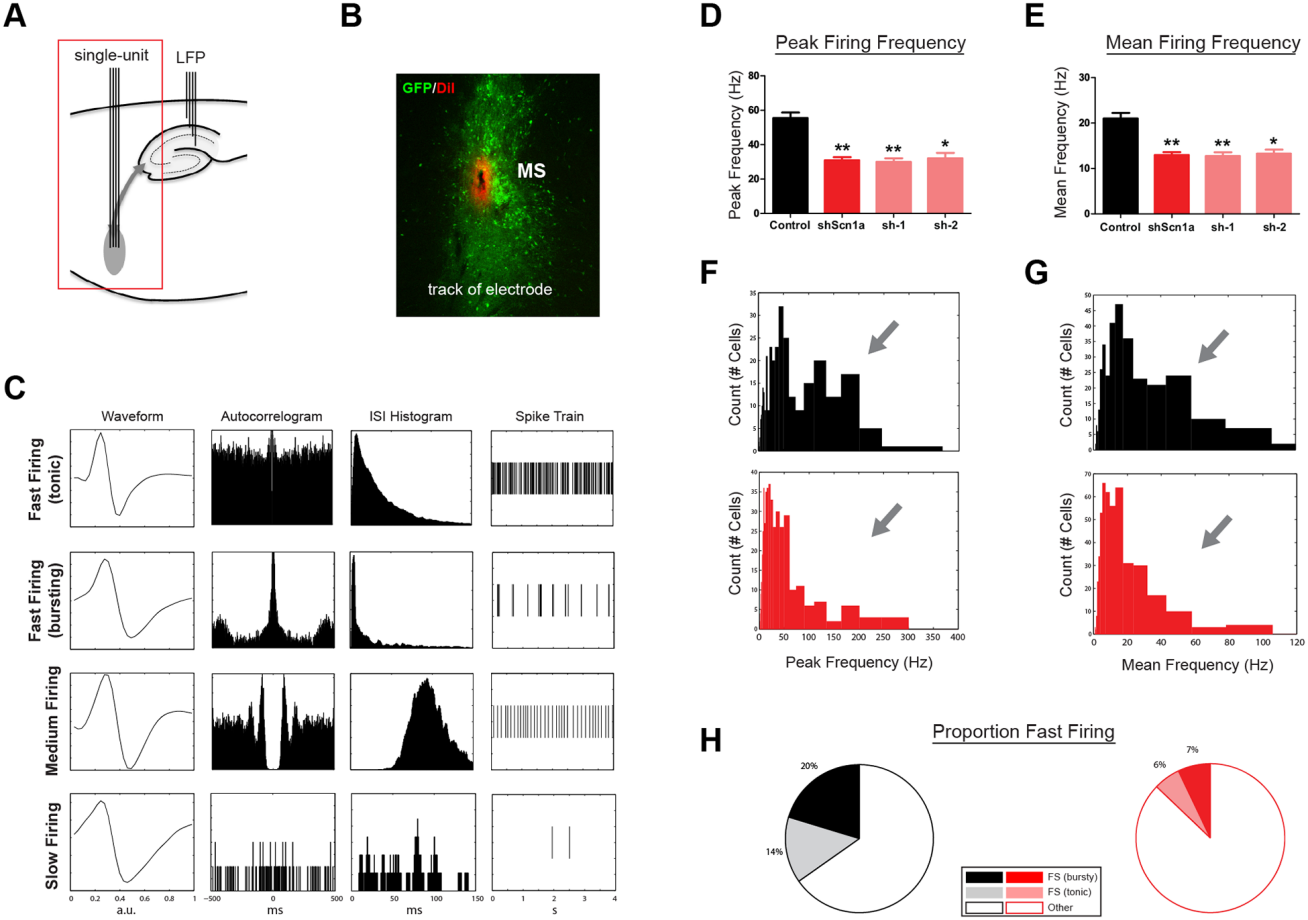
Reduction in fast- and burst-firing neurons after MSDB knockdown of Na_v_1.1. (A) Schematic of electrode recording sites. Single-units were recorded after lentiviral injections in the MSDB. (B) Example of electrode track (DiI) located in the MSDB injection site (GFP). (C) Examples of single-units recorded in the MSDB. Each row represents one unit, and each column shows a different type of plot for that unit (average waveform, autocorrelogram, inter-spike-interval histogram, and example of spike train). Multiple firing types were observed, including fast-firing (rhythmic bursting and tonic), medium-firing, and slow-firing. (D,E) Average peak and mean firing frequency for all units recorded in control or shScn1a groups (‘shScn1a’ refers to sh-1 & sh-2 pooled data). Bars represent mean +/- SEM. *p<.05, **p<.01. (F,G) Histograms of peak and mean firing frequency for all units (controls, black; shScn1a, red). Units with high peak frequencies were markedly reduced in shScn1a rats. (H) The proportion of units with fast- and burst-firing characteristics was also significantly lower (p<.01).

We next performed acute stereotaxic recordings *in vivo* in urethane-anesthetized rats to determine the impact of Na_v_1.1 loss of function on MSDB neuronal firing properties. Single-unit electrodes were lowered into the MSDB, and LFP electrodes were placed in the dorsal hippocampus CA1 region (Fig 3A). Single units were recorded incrementally along the dorsal-ventral axis of the MSDB (also see Materials and Methods). Only recordings from electrodes that passed through the infected, GFP-expressing, region were analyzed (Fig 3B). In 13 rats (4 control, 4 sh-1, 5 sh-2), 791 units were recorded. We observed a variety of firing patterns among MSDB units. Many exhibited fast-firing characteristics, while others fired at moderate or slow frequencies (Fig 3C). After knockdown of Na_v_1.1, we observed a significant reduction in both the peak (control 55.5 +/- 3.2 Hz, shScn1a 31.0 +/- 1.8 Hz, mean +/- SEM; t(782) = 7.17, p<.01) and mean firing frequencies (control 21.0 +/- 1.2 Hz, shScn1a 12.9 +/- 0.6 Hz, mean +/- SEM; t(789) = 6.42, p<.01; Fig 3D and 3E). Importantly, both sh-1 and sh-2 vectors produced the same effect (peak freq: F(3,1239) = 24.68, p<.01; mean freq: F(3,1253) = 20.52, p<.01), lending confidence that these changes were the result of knockdown of our target gene rather than an off-target effect.

Interestingly, there was a strong alteration of MSDB unit firing frequency histograms (Fig 3F and 3G). While control cells segregated into two populations, one with peak frequencies greater than 75 Hz and one with peak frequencies below 75 Hz (Fig 3F), very few cells fell into the former category after knockdown of Na_v_1.1. Rather, cells with lower peak frequencies represented the majority of the population. The distribution of these populations differed significantly between groups (KStest = 0.25, p<.01). Since the property of having a high peak firing frequency is characteristic of units with fast- and burst-firing patterns, we quantified the proportion of neurons in each of these categories (see Materials and Methods and S2B Fig). In agreement with previous reports, we observed firing patterns that have been previously associated with specific cell types [43]. In controls, some neurons exhibited fast-firing characteristics (34%), typical of GAD67- or PV-expressing neurons. These included neurons with tonic- (14%) and burst-firing patterns (20%). Other neurons (66%) fired at moderate or slow frequencies (Fig 3C). However, after knockdown of Na_v_1.1, the proportion of neurons with fast- (6%) and burst-firing (7%) types was significantly reduced (*X*^2^(2) = 53.9, p<.01; Fig 3H). Together, these results suggest that Na_v_1.1 is critical for supporting high frequency firing in neurons.

### Dysregulation of Hippocampal Theta Rhythm

**Fig 4:**
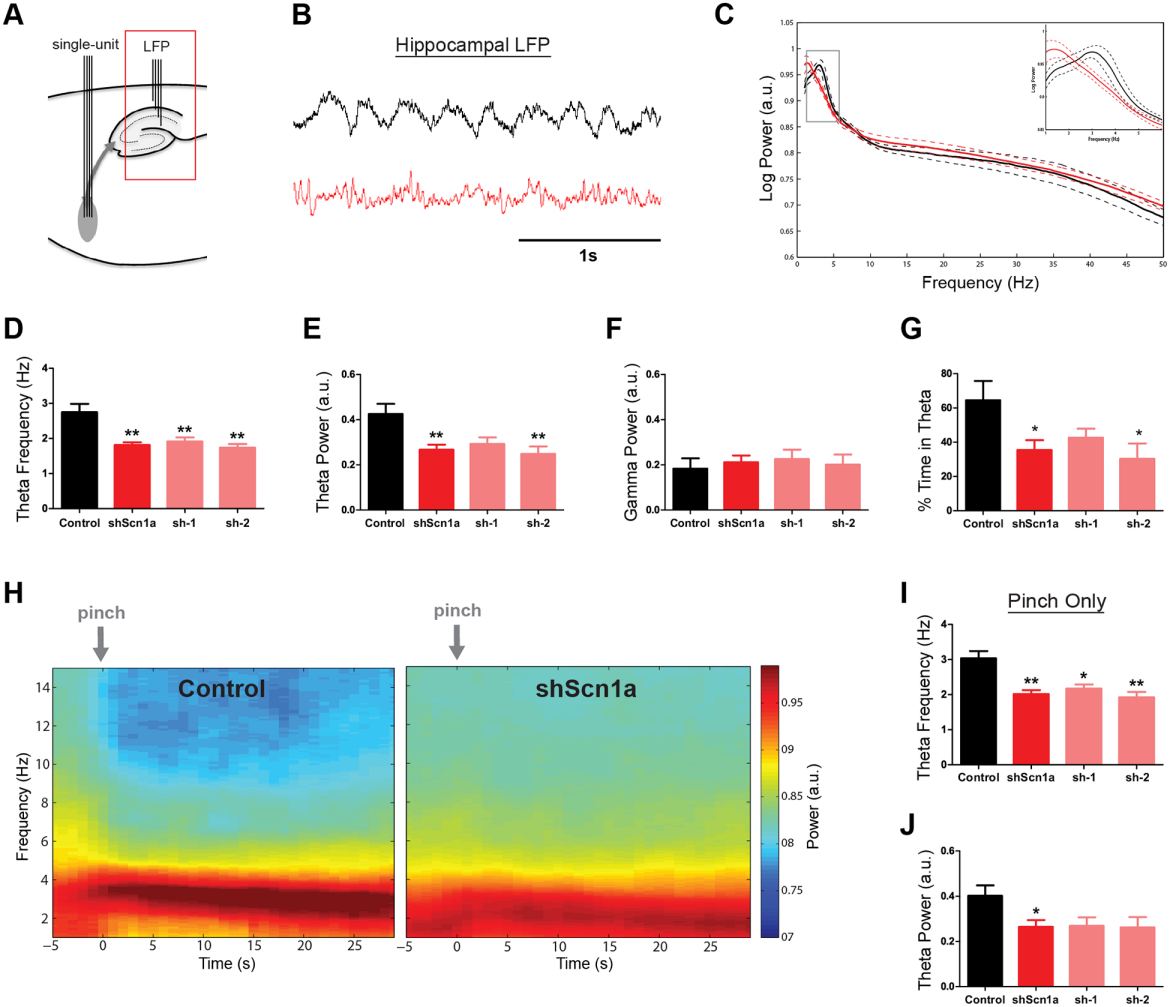
Suppression of hippocampal theta rhythm after MSDB Na_v_1.1 knockdown. (A) Schematic of recording site. (B) Example of LFP signal from the dorsal hippocampus of a control (black) and shScn1a (red) rat. (C) Group averaged power spectra for control and shScn1a rats. Inset shows power in the theta band. Solid line indicates mean, dashed line indicates SEM. (D-F) Theta frequency and theta power were significantly reduced in shScn1a rats, but no change in high frequencies in the gamma band were observed. (G) shScn1a rats also spent less time in theta. (H) Group averaged time-frequency spectrograms for 30s of LFP data time-locked to tail-pinch events. Notice that a prominent theta rhythm is apparent in response to the tail pinch but occurs at a lower frequency in shScn1a rats. (I,J) Quantification of theta frequency and theta power for data in panel H. Bars represent mean +/- SEM. *p<.05, **p<.01.

We next investigated the downstream consequence of MSDB Na_v_1.1 knockdown on hippocampal LFP activity (Fig 4A and 4B and S3A and S3B Fig). On average, we observed a significant reduction in hippocampal theta power (control 0.42 +/- 0.04 a.u., shScn1a 0.27 +/- 0.02 a.u., mean +/- SEM; t(16) = 3.52, p<.01) and frequency (control 2.75 +/- 0.23 Hz, shScn1a 1.81 +/- 0.08 Hz, mean +/- SEM; t(4.74) = 4.74, p<.01) after MSDB knockdown of Na_v_1.1 (Fig 4B–4E). Both the sh-1 and sh-2 vectors produced a similar reduction in theta properties (theta power: F(2,15) = 6.41, p<.01; theta freq: F(2,15) = 11.61, p<.01; Fig 4D and 4E). In contrast, no change in higher frequencies in the gamma range was observed (t(16) = 0.53, p>.05; Fig 4C and 4F). Notably, shScn1a rats also spent less time in theta on average (Controls 64.5% +/- 11.2%, shScn1a 35.5% +/- 5.7%, Mean +/- SEM; t(16) = 2.59, p<.05; Fig 4G).

Under urethane anesthesia, theta rhythm is observed both spontaneously and in response to somatosensory stimulation. Therefore, the tail of the rat was pinched periodically during each recording session (3 times per session; S3C and S3E Fig; also see Materials and Methods). Analysis of the LFP data time-locked to the tail pinch (Fig 4H–4J and S3D Fig) also revealed a clear reduction in the frequency of theta oscillations (t(12) = 4.65, p<.01; Fig 4I), with a moderate reduction in power (t(12) = 2.63, p<.05; Fig 4J). Notably, shScn1a rats spent less time in theta during the 30 seconds following the tail pinch (Controls 84.7% +/- 6.7%, shScn1a 49.7% +/- 8.4%, Mean +/- SEM; t(12) = 3.09, p<.01). We also performed a separate analysis of theta properties restricted to automatically-detected theta epochs (S3F Fig; see ‘theta detection’ in Materials and Methods). Confirming our results, there was a significant reduction in theta frequency (t(16) = 2.57, p<.05) and power (t(16) = 4.28, p<.01), and this effect was not sensitive to adjustments in the theta detection threshold (S3G Fig). Therefore, shScn1a rats spent less time in theta than controls, and when theta did occur, it exhibited a lower frequency.

### Reduced Theta Phase-locking of MSDB Units

**Fig 5:**
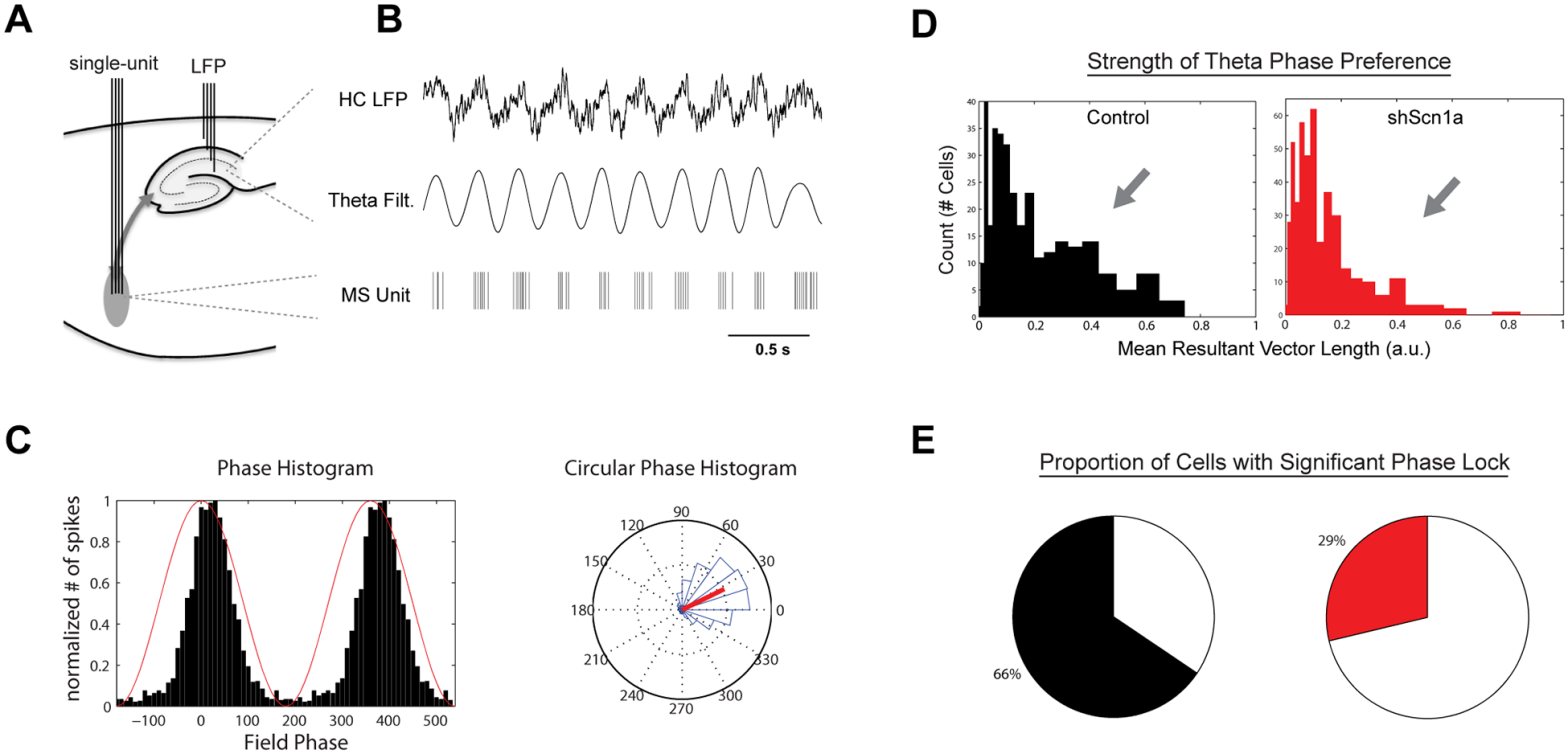
Reduced phase-locking of MSDB units to hippocampal theta rhythm. (A,B) Example of hippocampal LFP and theta-filtered signal with the simultaneously-recorded spike train from one MSDB unit. Notice the alignment (phase-locking) of spikes near the peak of the hippocampal theta oscillation. (C) Example of phase histogram of spikes in a standard (left) and rose plot (right) showing a strong theta phase preference. In left plot, red line indicates phase of hippocampal theta, and black bars indicate the phase of spikes. In right plot, phase histogram is shown in blue, theta phase as coordinates on the rose plot, and the red line indicates the mean resultant vector. (D) Histograms of theta phase-locking strength (mean resultant vector length) for units from controls and shScn1a rats. Phase-locking strength and (E) the proportion of units with statistically-significant phase-locking (Rayleigh test) were reduced after knockdown of Na_v_1.1 (p<.01).

The MSDB is believed to serve as a pacemaker for hippocampal theta oscillations by synchronizing hippocampal activity to a population of rhythmically-firing MSDB neurons. Evidence for this includes a prominent phase-locking of MSDB neurons to hippocampal theta rhythm [31–33,43]. To determine the impact of Na_v_1.1 downregulation on theta phase-locking of MSDB neurons, hippocampal theta epochs were extracted using an unbiased detection algorithm (Materials and Methods; S3F Fig), and the relationship of unit firing to theta phase was quantified (Fig 5A–5C). In control rats, we observed a distinct population of units (27%) with strong theta phase-locking (mean vector length > 0.2; Fig 5D). After Na_v_1.1 knockdown, the proportion of units with strong phase-locking (14%) was significantly reduced (*X*^2^(1) = 19.52, p<.01). The distributions of phase-locking strength also differed significantly between groups (KStest = 0.14, p<.01; Fig 5D). Similarly, the proportion of all units that reached a statistically-significant level of phase-locking (Rayleigh test) was greater in controls (66%) than in sh*Scn1a* knockdown animals (29%; *X*^2^(1) = 102.6, p<.01; Fig 5E). Thus, MSDB knockdown of Na_v_1.1 reduced the phase-locking of MSDB neurons to hippocampal theta oscillations.

As has been observed previously, the population of theta phase-locked neurons in the MSDB includes neurons with fast- and burst-firing properties [31,32,43]. It is therefore likely that the pronounced reduction in peak firing frequencies observed in MSDB neurons (Fig 3F–3H) contributed to the reduction in the population of theta phase-locked neurons after knockdown of Na_v_1.1. We found that the proportion of significantly phase-locked neurons was reduced among units characterized by both a high peak firing frequency (>75 Hz; *X*^2^(1) = 10.81, p<.01) and lower peak firing frequency (<75 Hz; *X*^2^(1) = 32.12, p<.01; S4 Fig). Thus, the reduction in the population of neurons with a peak frequency greater than 75 Hz (Fig 3F) indeed contributed to the overall reduction in theta phase-locking, but not exclusively, as the proportion of significantly phase-locked neurons was also reduced among units with relatively slower peak frequencies.

### Impaired Spatial Working Memory

We next investigated how knockdown of Na_v_1.1 in the MSDB affected cognitive function on spatial navigation tasks. Rats were first tested in an open field to observe voluntary exploratory behavior. There was no difference in running speed (t_(22)_ = 0.79, p>.05) or total distance explored (t(22) = 0.79, p>.05) between the control and shScn1a groups (S5A and S5B Fig). There was a small decrease in the time shScn1a rats spent exploring the center of the arena (t(22) = 2.25, p<.05; S5C Fig), indicating a modest increase in anxiety-like behavior [44].

Rats were then tested in the Morris water maze. During the first Habituation session, there was no difference in the mean swim speed (t(22) = 1.01, p>.05), total distance explored (t(22) = 1.0, p>.05), or the mean distance from the center (t(22) = 0.83, p>.05) between control and shScn1a rats (S5D–S5F Fig). Thus, both groups explored their environment equally in the water maze, with no signs of center-avoidance behavior. In subsequent acquisition and probe trials, there was also no difference between groups in latency to find the platform (F(1,22) = 0.037, p>.05, effect by group, two-way repeated-measures ANOVA; S5G Fig) or time spent in the target quadrant (t(22) = 1.31, p>.05; S5I Fig), indicating that spatial reference memory was intact. However, subtle differences in behavior did emerge. The path efficiency to find the platform during acquisition trials was significantly worse (F(1,22) = 5.03, p<.05, effect by group, two-way repeated-measures ANOVA; S5H Fig), and the number of times the rats crossed over the platform location during the probe session was lower (t(22) = 2.54, p<.05; S5J Fig) in shScn1a-treated rats. Furthermore, on the reversal session, where the platform was moved to a new location, there was an even more pronounced effect on path efficiency (F(1,22) = 10.22, p<.01 effect by group, F(1,22) = 5.26, p<.01 interaction, two-way repeated-measures ANOVA; S5K and S5L Fig), suggesting that short-term memory processes might be affected.

**Fig 6:**
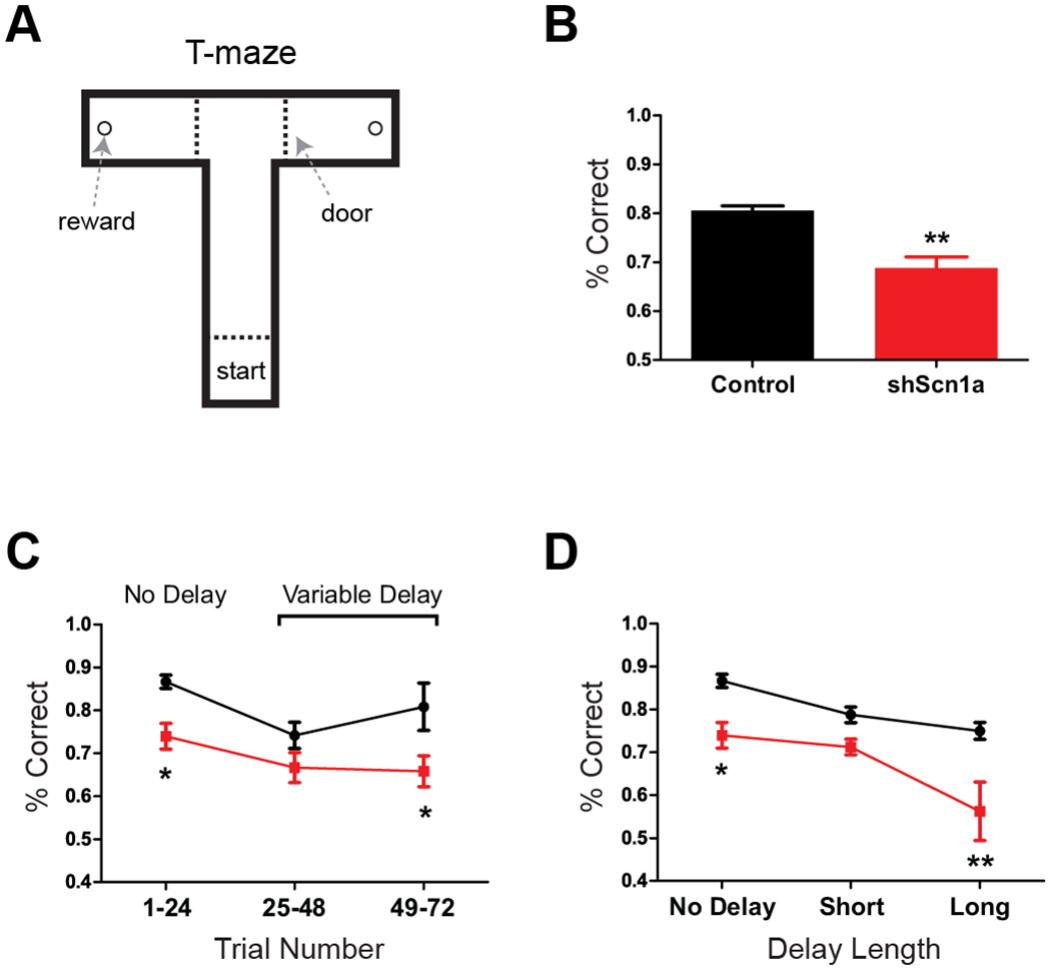
Impaired spatial working memory on a T-maze after MSDB Na_v_1.1 knockdown. (A) Schematic of T-maze apparatus used for working memory task. Rats were trained to run from the start box to the end arm to receive a food reward. On each test trial, rats were given a Sample run (a forced turn either left or right), followed by a variable delay period, and then a Choice run. Correct choices required the rat to remember and recall the direction it had travelled on the Sample run and to then choose the opposite direction on the Choice run. (B) Mean choice accuracy for all trials was significantly worse with knockdown of Na_v_1.1 compared to controls (p<.01). (C) Choice accuracy segregated into 24-trial blocks. Performance was initially lower for both groups during ‘variable delay’ trials, but performance improved in controls and not in shScn1a rats. (D) Choice accuracy segregated by delay length (short, 15-30s; long, 60s). While performance was lower overall for shScn1a rats, they were substantially worse at the longest delay, performing no better than chance levels. Data show mean +/- SEM. *p<.05, **p<.01.

We therefore tested spatial working memory performance using a rewarded alternation (non-match-to-place) protocol in a T-maze (Fig 6A; also see Materials and Methods). Overall, rats performed significantly worse after knockdown of Na_v_1.1, with a choice accuracy of only 68.8% +/- 2.3% compared to 80.6% +/- 1.0% in controls (t(8) = 4.67, p<.01; Fig 6B). Furthermore, performance in controls improved during the variable delay trials, but performance in the shScn1a group did not (F(1,8) = 21.8, p<.01 effect by group, two-way repeated-measures ANOVA with Bonferonni post-test for trial block; Fig 6C). Interestingly, while performance in both groups decreased as a function of the delay (F(1,8) = 11.0, p<.01 effect of delay, two-way repeated-measures ANOVA), performance in shScn1a rats was substantially worse for the longest (60s) delay period (F(1,8) = 17.7, p<.01 effect by group, two-way repeated-measures ANOVA with Bonferronni post-test for delay length), with a choice accuracy no better than chance levels (56.3% +/- 6.8%; t(4) = 0.91, p>.05, one-sample t-test; Fig 6D). Together, these results point to a significant working memory deficit after MSDB knockdown of Na_v_1.1.

### T-maze Performance is Associated with Dynamic Changes in Theta Frequency

**Fig 7:**
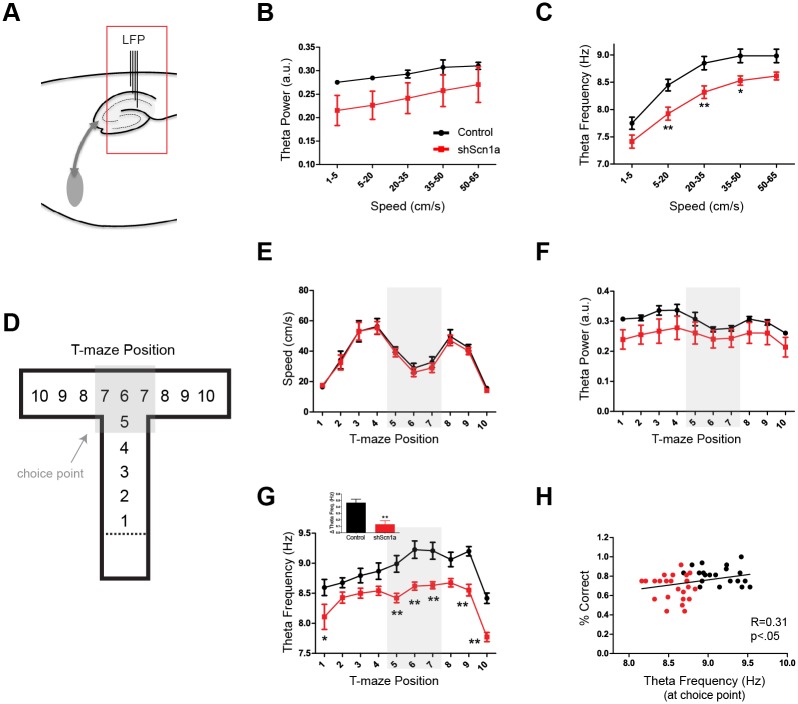
T-maze performance is associated with dynamic, speed-independent regulation of hippocampal theta frequency at the choice point. (A) Schematic of recording site. (B-C) Average theta power and frequency during navigation in the T-maze as a function of running speed. (B) Theta power was related to speed (p<.01) and not significantly different between groups (p>.05). (C) Theta frequency was also related to speed (p<.01), but was significantly slower in shScn1a rats (p<.01). (D-H) The maze was divided into 10 position bins to investigate dynamic changes in theta oscillations during behavior. (E) As the rats navigated down the maze, running speed followed a predictable pattern (increasing in the central arm, decreasing at the choice point, and increasing again in the end arm). There was no difference in running speed between groups (p>.05). (F) Theta power followed a similar pattern to running speed and did not differ significantly between groups (p>.05). (G) In contrast, theta frequency dissociated from its relationship with speed at the choice point, evidenced by an increase in the frequency when running speed decreased. Interestingly, this increase in theta frequency was suppressed by knockdown of Na_v_1.1 (p<.01). Inset shows change in theta frequency at choice point compared to central arm. Group data show mean +/- SEM. *p<.05, **p<.01. (H) The frequency of theta rhythm at the choice point was significantly correlated with choice accuracy (r = 0.31, p<.05). Each point represents one session (segregated by delay length) for a given rat.

Rats that navigated the T-maze were implanted with depth electrodes in the hippocampal CA1 region, allowing us to assess LFP activity during performance on this working memory task (Fig 7A and S6A and S6B Fig). On average, both the power (F(1,7) = 13.32, p<.01 effect by speed, two-way repeated-measures ANOVA; Fig 7B) and frequency (F(1,7) = 199.3, p<.01 effect by speed; Fig 7C) were related to running speed. However, the frequency of theta rhythm was significantly slower after knockdown of Na_v_1.1 (controls 8.60 +/- 0.11, shScn1a 8.16 +/- 0.10, Mean +/- SEM; F(1,7) = 9.59, p<.05 effect by group; Fig 7C and S6B and S6C Fig), consistent with the reduction in theta frequency observed in urethane-anesthetized animals. There was also a trend towards a reduction in theta power but this effect was not significant (F(1,7) = 1.90, p>.05 effect by group; Fig 7B), and there was no effect on gamma activity (slow gamma, t(7) = 0.83, p>.05; fast gamma, t(7) = 0.95, p>.05). Thus, Na_v_1.1 knockdown in the MSDB produced a selective decrease in hippocampal theta frequency in freely-behaving rats.

To investigate how hippocampal theta oscillations were related to behavior in the T-maze, we divided the maze into 10 position bins (Fig 7D). As the rats navigated down the maze, running speed followed a predictable pattern, increasing in the central arm, then decreasing at the choice point, and finally increasing again in the end arm (Fig 7E). Notably, there was no difference in running speed between groups (F(1,7) = 0.14, p>.05 effect by group, two-way repeated-measures ANOVA). Theta power followed an almost identical pattern to running speed (Fig 7F), indicating that the power was coupled to speed during navigation in the maze. There was also no significant group difference in theta power as a function of position (F(1,7) = 1.61, p>.05 effect by group). In contrast, theta frequency dissociated from its relationship with speed at the choice point, as evidenced by an increase in the frequency precisely when running speed decreased (Fig 7G and S6E Fig). Therefore, the dynamic changes in theta frequency, but not power, were related to behavior. Moreover, the increase in theta frequency observed at the choice point was absent after knockdown of Na_v_1.1 in the MSDB (F(1,7) = 13.6, p<.01 effect by group, F(1,7) = 2.93, p<.01 effect of interaction, F(1,7) = 36.9, p<.01 effect by position, two-way repeated-measures ANOVA with Bonferonni post-test by position), and there was a significant positive correlation between choice accuracy and theta frequency at the choice point (r = 0.31, p<.05; Fig 7H).

## Discussion

Our results show for the first time that loss of function of Na_v_1.1 impairs fast- and burst-firing properties of neurons *in vivo*. In the MSDB, the proportion of neurons that fire phase-locked to hippocampal theta oscillations was reduced, and the ability of the MSDB to properly regulate theta rhythm was disrupted. As a result, a prominent slowing of hippocampal theta rhythm was observed in both urethane-anesthetized and freely-behaving rats. MSDB knockdown of Na_v_1.1 impaired spatial working memory in a T-maze task, but left spatial reference memory relatively intact. In controls, theta frequency increased at the choice point of the maze despite a reduction in running speed, but this pattern was absent in shScn1a rats. Moreover, theta frequency at the choice point was correlated to task performance, demonstrating a link between theta frequency dysregulation and working memory impairment. Together, these findings suggest that loss of function of Na_v_1.1 leads to significant disruption in theta rhythm. Such temporal patterns are critically linked to information processing, and as shown here in the septo-hippocampal network, deficits may be related to impairments in cognition.

### Role of Na_v_1.1 in MSDB neurons

Multiple cell types have been described in the MSDB, including GABAergic neurons with fast- and burst-firing properties and cholinergic neurons exhibiting a slow-firing phenotype [43,45,46]. In addition, a glutamatergic population projects to the hippocampus and contains neurons with slow- and cluster-firing properties [46,47]. Here we report Na_v_1.1 expression in both cholinergic and GABAergic neurons. Although Na_v_1.1 expression has been primarily associated with GABAergic neurons in cortical regions [27,40], recent evidence demonstrates that some forebrain excitatory neurons express Na_v_1.1 [41], as do serotonergic and cholinergic neurons in sub-cortical regions [42]. Interestingly, the impact of Na_v_1.1 knockdown in this study was predominantly on a population of fast- and burst-firing neurons, which are likely to be GABAergic but not cholinergic [43]. This is best shown by the histograms in Fig 3F and 3G, which are overlapping in the slow (but not high) frequencies when comparing between groups. Therefore, although we cannot rule out a possible effect on slow-firing cells, the evidence indicates that Na_v_1.1 is critical for neurons to support high-frequency firing, and while Na_v_1.1 may contribute to the sodium channel composition in multiple cell types, it is primarily in those neurons with fast-spiking behavior that functional deficits are evident—suggesting that reduced Na_v_1.1 expression effectively acts as a low-pass filter on spike output.

### MSDB Regulation of Hippocampal Theta Rhythm

An important function of MSDB neurons is to modulate hippocampal theta oscillations. The MSDB receives ascending input from the brainstem, suprammamillary nucleus and hypothalamus, which activates MSDB neurons [30,48,49]. The cholinergic and glutamatergic projection neurons excite hippocampal networks and, in combination with inputs from the entorhinal cortex, provide the excitatory drive necessary to activate or augment hippocampal theta oscillations. MSDB GABAergic projection neurons, on the other hand, are positioned to serve as pacemakers for theta oscillations, as they innervate local interneurons throughout the hippocampal formation and thereby control the timing of pyramidal cell firing through synchronized disinhibition [30,32,33,36,50,51]. Thus, phasic input from GABAergic, fast- and burst-firing, neurons are likely critical for modulating the frequency of theta rhythm. This idea is consistent with our observation that a specific reduction in MSDB fast-firing neurons after Na_v_1.1 knockdown led to a selective dysregulation of theta frequency.

Several lines of evidence suggest that theta phase-locked neurons in the MSDB have an important and primary role in regulating hippocampal theta oscillations [32,52–54], and that the expression of theta activity in the hippocampus is, in fact, related to the proportion of active theta-rhythmic MSDB neurons [48,55]. In this study, we found that the proportion of significantly phase-locked neurons was reduced by almost half in shScn1a rats. Therefore, the reduction in this theta-rhythmic population likely contributed to the dysregulation of hippocampal theta oscillations in shScn1a rats, and suggests that Na_v_1.1 is important to support neuronal firing patterns involved in rhythmic activity.

### Effects on Learning and Memory

The MSDB is essential for learning and memory processes, as lesions or inactivation of the MSDB generates profound deficits in a variety of memory tasks [34,56–59]. In this study, MSDB knockdown of Na_v_1.1 led to a specific working memory deficit, leaving reference memory relatively intact. This is reminiscent of a series of studies that used neurotoxins to selectively damage specific MSDB neuronal populations. These studies demonstrated that lesions to the GABAergic, but not cholinergic, population of MSDB neurons also produced deficits in working, but not reference, memory [60,61]. Therefore, the fact that the deficit observed following Na_v_1.1 knockdown was specific to working memory is consistent with our electrophysiological evidence indicating that Na_v_1.1 knockdown predominantly impairs a population of putative GABAergic, fast- and burst-firing neurons.

One mechanism by which the MSDB engages in learning and memory processes is through the modulation of hippocampal theta oscillations. Loss of theta rhythm is associated with memory impairment [34,35,37,62], and involvement of human theta activity in cognitive function has been observed in a variety of tasks [3–6]. Here we found a relationship between the frequency of theta oscillations and behavior in a T-maze working memory task. Specifically, suppression of theta frequency after MSDB knockdown of Na_v_1.1 was associated with impaired performance on this task. These results agree with prior findings from our laboratory demonstrating that precise coordination of theta frequency is important for working memory processes [63].

In addition, we found that theta frequency changed dynamically during navigation in the T-maze, dissociating from its relationship with speed at the choice point, precisely when the rats had to decide to go left or right to receive the reward. Moreover, theta frequency at the choice point was positively correlated with choice accuracy, suggesting that speed-independent coordination of theta frequency is important for making the correct decision. These findings are supported by recent evidence demonstrating a role for theta rhythm in synchronizing neural activity between the hippocampus and neocortex at decision points [64,65]. It is reasonable to hypothesize that suppression of theta frequency in shScn1a rats may also have impaired the synchronization of the hippocampus with the neocortex, thereby contributing to the overall performance deficit. Together, our results suggest that the proper coordination of theta *frequency* is essential for learning and memory processes, that the MSDB is critically involved in this coordination, and that Na_v_1.1 is required for the MSDB to do this effectively.

### Implications

Taken together, these data have important implications for the role of Na_v_1.1 deficits in neurocognitive disorders. Previous work has suggested that social and cognitive deficits in Na_v_1.1 channelopathies may be caused by an increased ratio of excitatory to inhibitory synaptic transmission [66]. Our results suggest that the temporal patterns play an important role. We propose that Na_v_1.1 is necessary to support the neural activity responsible for coordinating brain rhythms, and that this is likely related to the ability of Na_v_1.1 to facilitate fast- and burst-firing patterns in neurons. These temporal patterns are essential for information processing, and, as we show in the septo-hippocampal network, such deficits may contribute to impaired cognitive function. In patients with Dravet syndrome, altered oscillatory patterns have indeed been found, primarily characterized by a shift to slower frequencies [26], in agreement with our animal data. Altered oscillations have also been found in autism and Alzheimer’s disease, two disorders characterized by profound cognitive and behavioral impairments, and for which Na_v_1.1 deficits may play a role [11,12,67–70]. Restoring the integrity of these rhythmic patterns may, therefore, offer a potential therapeutic target for ameliorating cognitive deficits.

## Supporting Information

S1 Fig Na_v_1.1 is expressed in cholinergic and GABAergic neurons in the MSDB. (A) Immunofluorescence for Na_v_1.1 revealed strong expression throughout the MSDB. Scale, 200 μm. (B) Na_v_1.1 co-localized with cell-type specific markers for cholinergic (ChAT) and GABAergic (GAD67 and PV) neurons in the MSDB. Scale, 100 μm. (C) Negative controls for Na_v_1.1 immunofluorescence included sections incubated with primary antibody plus Na_v_1.1 control antigen and sections incubated without primary antibody. Scale, 100 μm.
(TIF)

S2 Fig Properties of MSDB single-units. (A) Schematic of recording setup. (B) Scatter plot of MSDB unit properties, showing peak firing frequency, mean firing frequency and action potential width. Each point is one unit (325 control, 466 shScn1a). Fewer neurons exhibited fast-firing characteristics in shScn1a rats. Region #1 indicates units with tonic-firing properties and region #2 indicates units with bursting properties (related to Fig 3H; also see Materials and Methods). The average action potential width was not different between groups (p>.05).
(TIF)

S3 Fig Analysis of hippocampal LFP under urethane. (A) Schematic of recording setup. (B) Example of electrode track (red) in hippocampal CA1 region. (C) Example of power spectrum and time-frequency spectrogram for one recording session from a control and shScn1a rat. White arrows indicate time of tail pinch. (D) Group average power spectra for 30s LFP data time-locked to the tail pinch. Solid line represents group mean; dashed lines represent SEM. Notice that theta occurs at a slower frequency in the shScn1a group. (E) Average pinch count per session for controls and shScn1a rats. Pinch frequency was not different between groups (p>.05). (F) Example of automatic theta detection algorithm applied to one recording session. Top plot shows the normalized theta power as a function of time with the horizontal line indicating the detection threshold. Bottom plot shows automatically-detected theta epochs (white bars) on top of spectral data. Automatically-detected theta epochs were used in phase-locking analysis and to calculate the total time spent in theta. Also see Materials and Methods. (G) Spectral properties were also re-evaluated during theta-only periods. Reductions in theta power and frequency persisted when only theta epochs were analyzed, and this effect was not sensitive to adjustments in the theta-detection threshold. Group data represent mean +/- SEM. *p<.05, **p<.01.
(TIF)

S4 Fig Reduced theta phase-locking in units with high and low peak firing frequencies. Considering that a profound reduction in the population of MSDB units with a high peak firing frequency was observed in shScn1a rats (Fig 3F), we questioned to what degree this effect contributed to the reduction in the population of theta phase-locked neurons. (A) Histograms of peak firing frequency (from Fig 3F) showing a population of units with high peak frequencies (>75 Hz) and a population with relatively lower peak frequencies (<75 Hz) in controls. Units with high peak frequencies were substantially reduced in shScn1a rats. (B) Histograms of theta phase-locking strength (mean vector length) separated for units with either high or lower peak frequencies. (C) The proportion of significantly phase-locked neurons was reduced among units characterized by both a high peak firing frequency (p<.01) and a relatively lower peak firing frequency (p<.01).
(TIF)

S5 Fig Behavioral effects in the Open Field and Morris Water Maze. (A-C) Analysis of distance explored, mean speed and time spent in center for the Open Field test. A modest reduction in the time spent in the center was observed in shScn1a rats (p<.05). (D-F) Analysis of distance explored, mean speed, and mean distance from center during the Habituation session in the Water Maze. (G) No differences by group were found in the latency to find the platform during acquisition trials. (H) Path efficiency was lower in shScn1a rats (p<.05). (I) Both groups spent the same time exploring in the target quadrant (quadrant 1) during a Probe session. (J) However, shScn1a rats made fewer platform crossings during the probe session (p<.05). (K-L) Although both groups quickly learned to find the platform in a Reversal session, path efficiency was significantly worse in shScn1a rats (p<.01). Group data represent mean +/- SEM. *p<.05, **p<.01.
(TIF)

S6 Fig Dysregulation of theta frequency in the T-maze. (A) Schematic of recording setup. (B) Examples of LFP signal recorded in the T-maze from a control and shScn1a rat showing prominent theta rhythm. (C) Power spectra (of whole trial) for examples in B showing a lower theta frequency in the shScn1a rat. (D) Diagram of T-maze layout with choice point highlighted in grey and directions of x and y coordinates indicated in lower right. (E) Example of time-frequency spectrogram centered at the choice point (highlighted in grey and white bar on spectrogram). Top plots show x and y coordinates of the rat position with running speed below. Notice how speed slows down as the rat enters the choice point, and it is precisely at this time that the frequency of theta rhythm (seen on the spectrogram) increases.
(TIF)

S1 Methods (DOCX)
