# Supplementary figures and images for: Cognitive Deficits Associated with Nav1.1 Alterations: Involvement of Neuronal Firing Dynamics and Oscillations
Source: PLoS One. 2016 Mar 15;11(3):e0151538. doi: 10.1371/journal.pone.0151538 (PMC4792481; doi:10.1371/journal.pone.0151538)

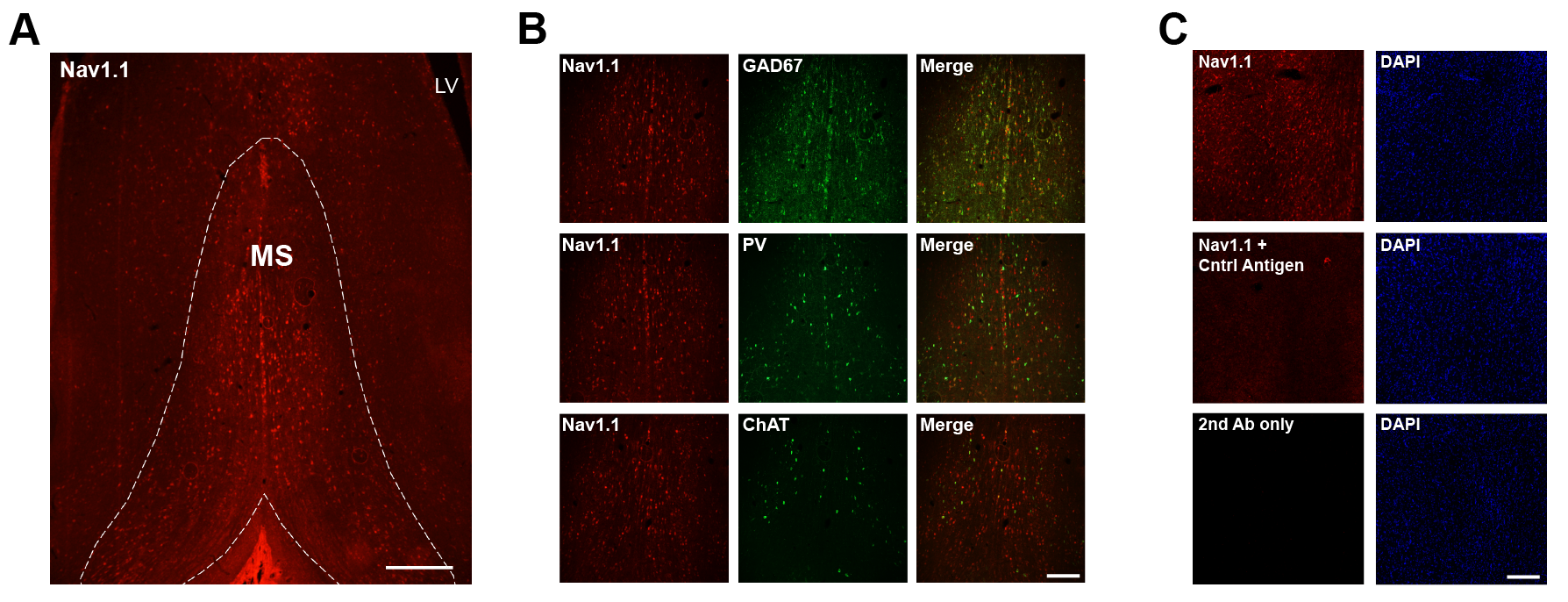

Supplement: S1 Fig — (A) Immunofluorescence for Nav1.1 revealed strong expression throughout the MSDB. Scale, 200 μm. (B) Nav1.1 co-localized with cell-type specific markers for cholinergic (ChAT) and GABAergic (GAD67 and PV) neurons in the MSDB. Scale, 100 μm. (C) Negative controls for Nav1.1 immunofluorescence included sections incubated with primary antibody plus Nav1.1 control antigen and sections incubated without primary antibody. Scale, 100 μm. (TIF) [file pone.0151538.s001.tif]

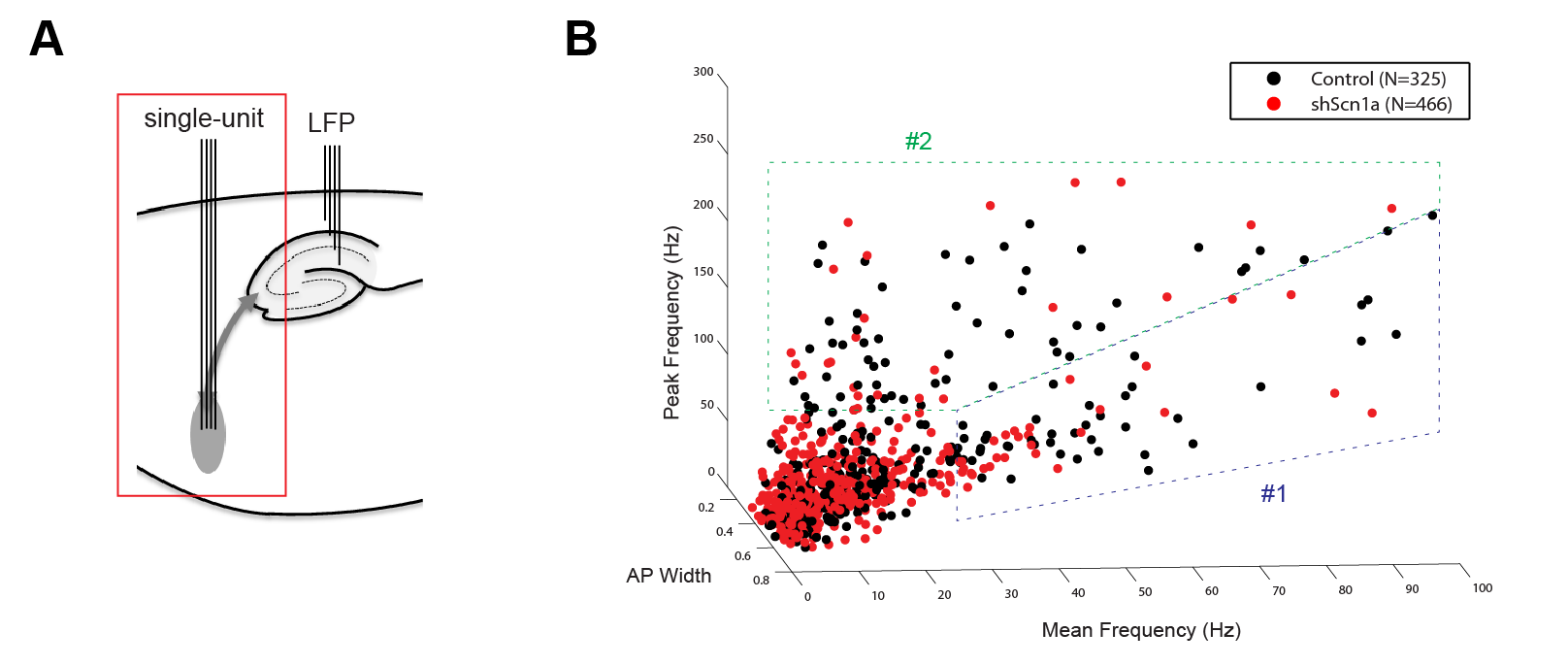

Supplement: S2 Fig — (A) Schematic of recording setup. (B) Scatter plot of MSDB unit properties, showing peak firing frequency, mean firing frequency and action potential width. Each point is one unit (325 control, 466 shScn1a). Fewer neurons exhibited fast-firing characteristics in shScn1a rats. Region #1 indicates units with tonic-firing properties and region #2 indicates units with bursting properties (related to Fig 3H; also see Materials and Methods). The average action potential width was not different between groups (p>.05). (TIF) [file pone.0151538.s002.tif]

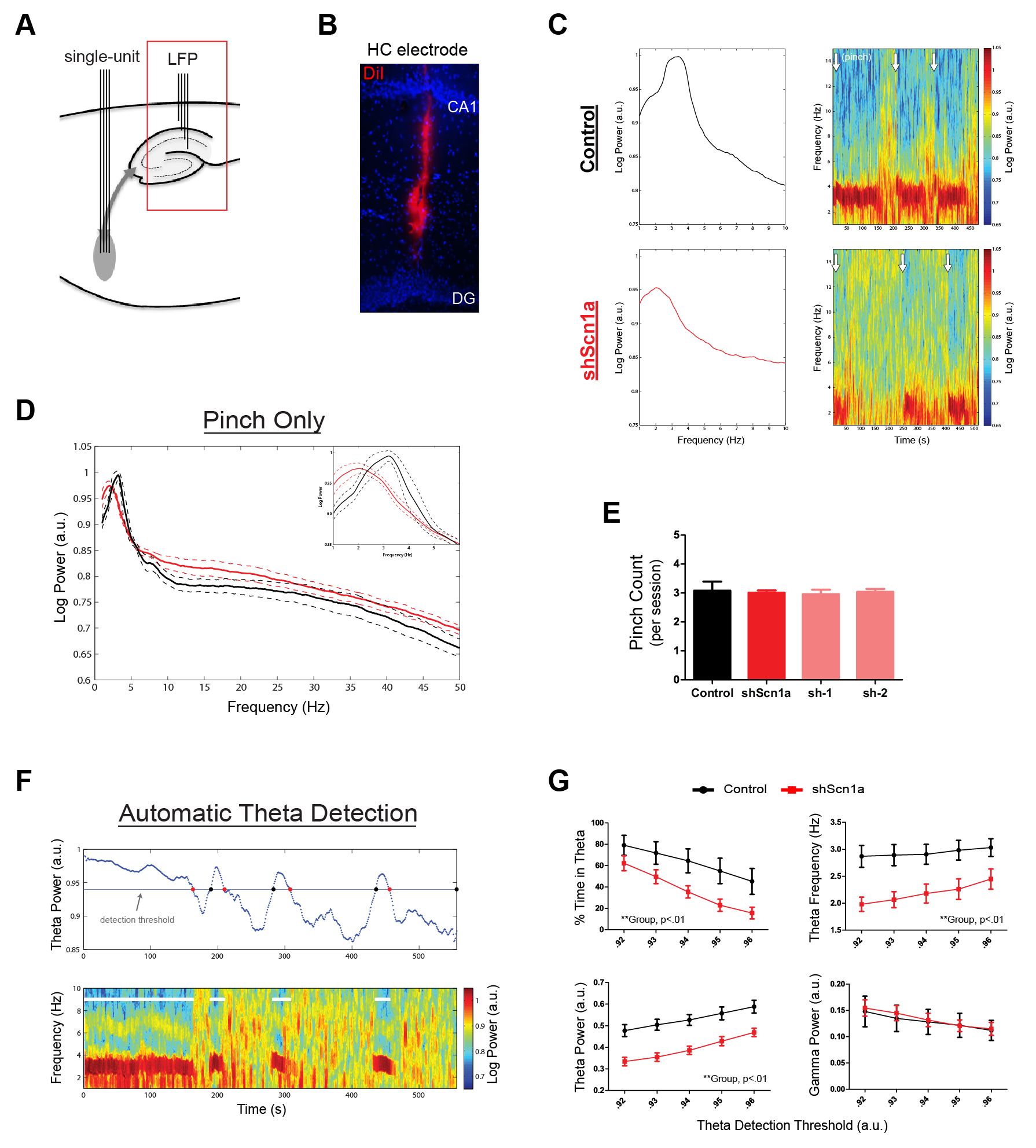

Supplement: S3 Fig — (A) Schematic of recording setup. (B) Example of electrode track (red) in hippocampal CA1 region. (C) Example of power spectrum and time-frequency spectrogram for one recording session from a control and shScn1a rat. White arrows indicate time of tail pinch. (D) Group average power spectra for 30s LFP data time-locked to the tail pinch. Solid line represents group mean; dashed lines represent SEM. Notice that theta occurs at a slower frequency in the shScn1a group. (E) Average pinch count per session for controls and shScn1a rats. Pinch frequency was not different between groups (p>.05). (F) Example of automatic theta detection algorithm applied to one recording session. Top plot shows the normalized theta power as a function of time with the horizontal line indicating the detection threshold. Bottom plot shows automatically-detected theta epochs (white bars) on top of spectral data. Automatically-detected theta epochs were used in phase-locking analysis and to calculate the total time spent in theta. Also see Materials and Methods. (G) Spectral properties were also re-evaluated during theta-only periods. Reductions in theta power and frequency persisted when only theta epochs were analyzed, and this effect was not sensitive to adjustments in the theta-detection threshold. Group data represent mean +/- SEM. *p<.05, **p<.01. (TIF) [file pone.0151538.s003.tif]

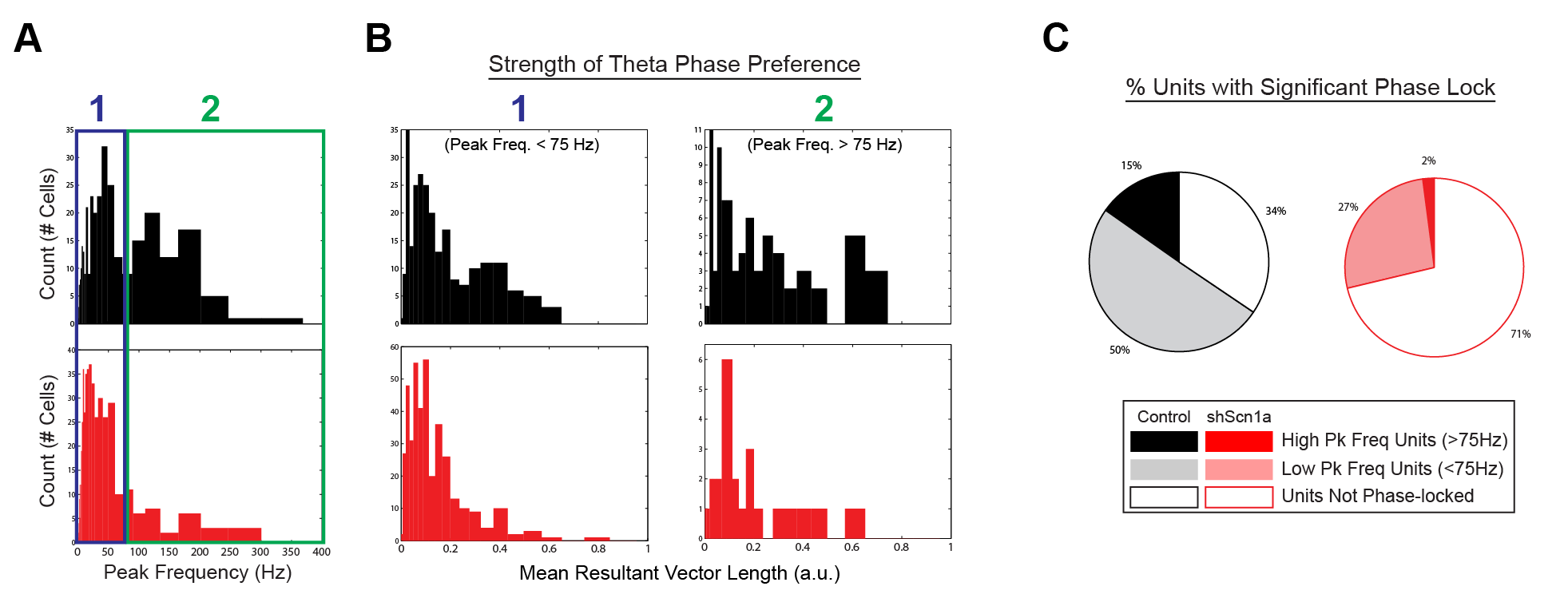

Supplement: S4 Fig — Considering that a profound reduction in the population of MSDB units with a high peak firing frequency was observed in shScn1a rats (Fig 3F), we questioned to what degree this effect contributed to the reduction in the population of theta phase-locked neurons. (A) Histograms of peak firing frequency (from Fig 3F) showing a population of units with high peak frequencies (>75 Hz) and a population with relatively lower peak frequencies (<75 Hz) in controls. Units with high peak frequencies were substantially reduced in shScn1a rats. (B) Histograms of theta phase-locking strength (mean vector length) separated for units with either high or lower peak frequencies. (C) The proportion of significantly phase-locked neurons was reduced among units characterized by both a high peak firing frequency (p<.01) and a relatively lower peak firing frequency (p<.01). (TIF) [file pone.0151538.s004.tif]

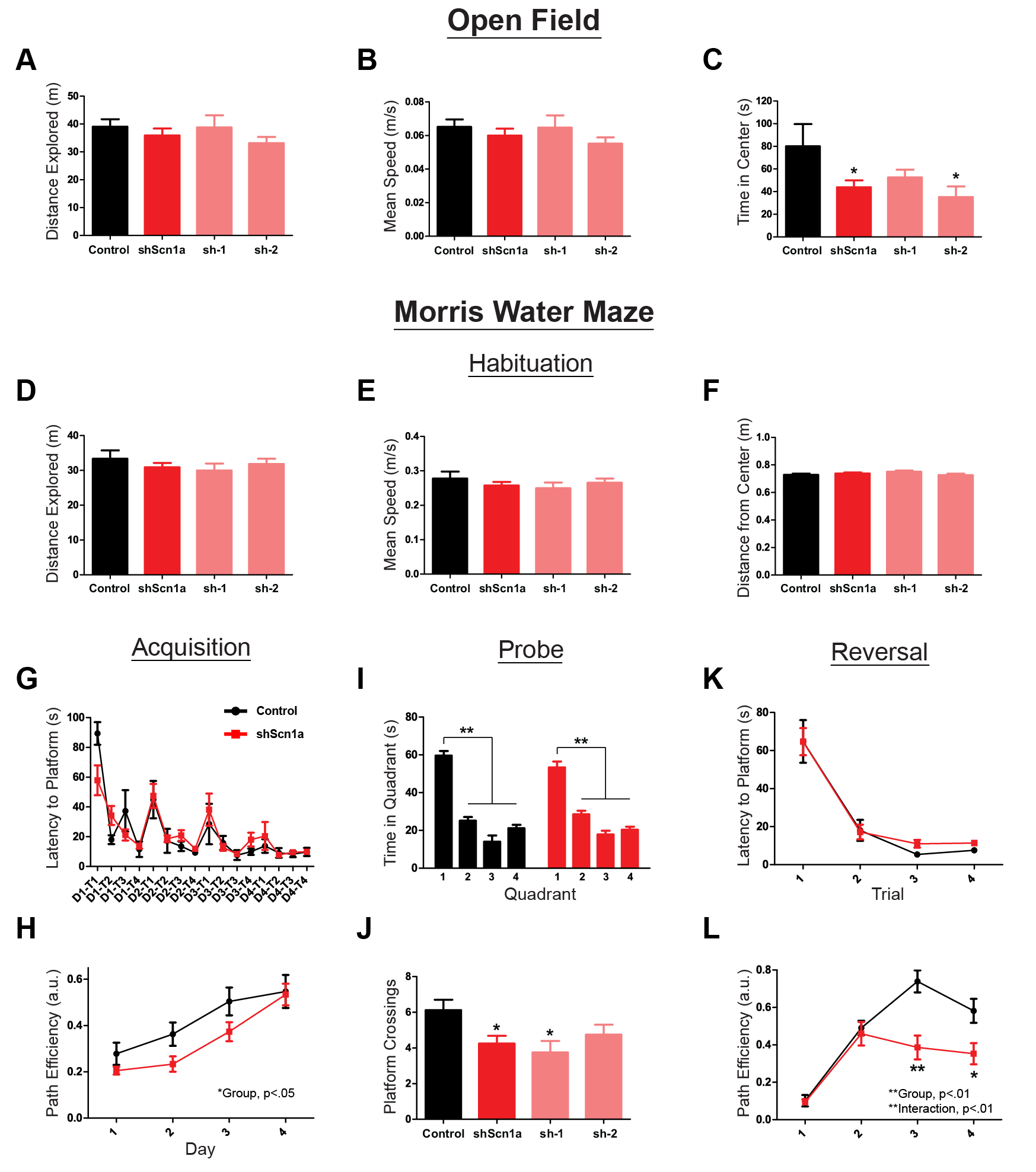

Supplement: S5 Fig — (A-C) Analysis of distance explored, mean speed and time spent in center for the Open Field test. A modest reduction in the time spent in the center was observed in shScn1a rats (p<.05). (D-F) Analysis of distance explored, mean speed, and mean distance from center during the Habituation session in the Water Maze. (G) No differences by group were found in the latency to find the platform during acquisition trials. (H) Path efficiency was lower in shScn1a rats (p<.05). (I) Both groups spent the same time exploring in the target quadrant (quadrant 1) during a Probe session. (J) However, shScn1a rats made fewer platform crossings during the probe session (p<.05). (K-L) Although both groups quickly learned to find the platform in a Reversal session, path efficiency was significantly worse in shScn1a rats (p<.01). Group data represent mean +/- SEM. *p<.05, **p<.01. (TIF) [file pone.0151538.s005.tif]

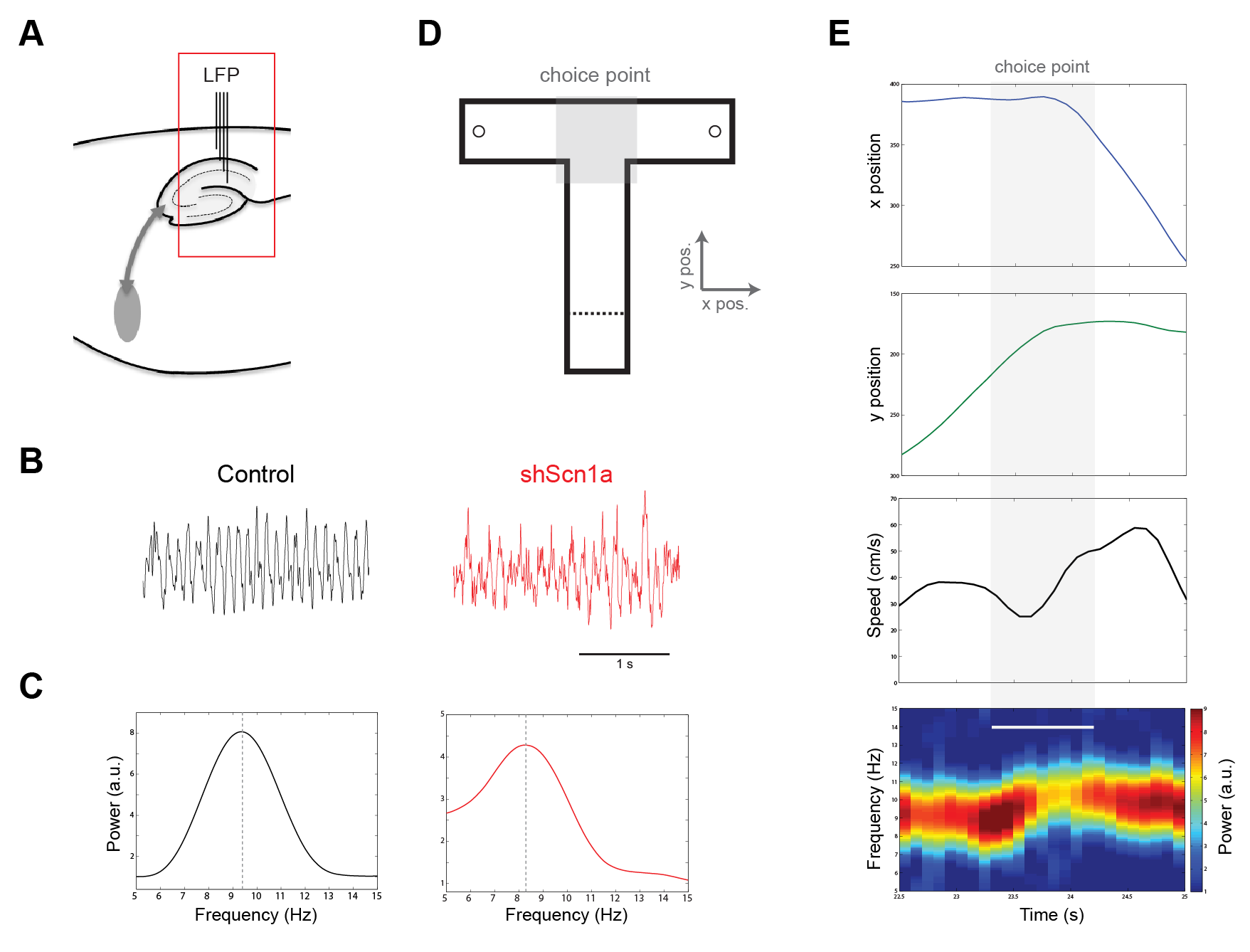

Supplement: S6 Fig — (A) Schematic of recording setup. (B) Examples of LFP signal recorded in the T-maze from a control and shScn1a rat showing prominent theta rhythm. (C) Power spectra (of whole trial) for examples in B showing a lower theta frequency in the shScn1a rat. (D) Diagram of T-maze layout with choice point highlighted in grey and directions of x and y coordinates indicated in lower right. (E) Example of time-frequency spectrogram centered at the choice point (highlighted in grey and white bar on spectrogram). Top plots show x and y coordinates of the rat position with running speed below. Notice how speed slows down as the rat enters the choice point, and it is precisely at this time that the frequency of theta rhythm (seen on the spectrogram) increases. (TIF) [file pone.0151538.s006.tif]
