# Supplementary material for: Cognitive Deficits Associated with Nav1.1 Alterations: Involvement of Neuronal Firing Dynamics and Oscillations
Source: PLoS One. 2016 Mar 15;11(3):e0151538. doi: 10.1371/journal.pone.0151538 (PMC4792481; doi:10.1371/journal.pone.0151538)
Supplement: S1 Methods — (DOCX) [file pone.0151538.s007.docx]

**S1 Methods**

**Animals**

Male, adult (postnatal day 60-180), Sprague-Dawley rats (Charles River Laboratories, Wilmington, MA) were used. Animals were housed in standard facilities under USDA- and AAALAC-approved conditions with a 12:12 hour light:dark cycle and ad libitum access to food and water (except when indicated otherwise for behavioral procedures). All procedures were approved by the Dartmouth College Institutional Animal Care and Use Committee and were performed in accordance with the Institute for Laboratory Animal Research (ILAR) Guide for the Care and Use of Laboratory Animals.

**Cloning and Viral Packaging**

Two shRNA sequences targeting the rat *Scn1a* gene were used (sh-1: 5’ - CCAGAGCGATTATGTGACAAGCATT - 3’; sh-2: 5’ - AAAGAGAAACTCAACGAAA - 3’). The target sequence for sh-1 was previously validated in our lab as an siRNA [1]. Both target sequences were evaluated for specificity using the Dharmacon database (Thermo Fisher Scientific Inc.). Each shRNA was cloned into the FUGW vector with a U6 promoter between the BstBI and PacI ligation sites. The FUGW vector, which also contains a Green Fluoresent Protein (GFP) expression sequence downstream of an ubiquitin promoter, has been published previously [2]. An FUGW vector containing the GFP expression sequence but no shRNA sequence was used as control.

The lentivirus was packaged by calcium phosphate-mediated transfection of HEK293 FT cells (Invitrogen). Cells were grown in Iscove’s modified Dulbeco’s medium (IMDM), high glucose (Invitrogen), containing 10% fetal bovine serum (FBS), 0.1 mM MEM nonessential amino acids, 2 mM L-glutamine and 1% penicillin/streptomycin, and plated one day prior to transfection at an approximate density of 3.5 x 10^4^ cells/cm^2^. In one polystyrene tube, 20 µg of the FUGW vector, 13 µg of pCMVΔ8.9 and 9 ug of pVSV-g (each maintained in TE at 1 ug/ul) were combined with 100 µl of 2.5 M CaCl_2_ and water to a total volume of 1 ml. This mixture was then added slowly to a second tube containing 1 ml of 2X HBS (281 mM NaCl, 50 mM HEPES, 1.5 mM Na2HPO4 heptahydrate monobasic, pH 7.0), and incubated for 30 min at room temperature in the dark. 1 ml of this mixture was then added to each 10 cm plate of HEK293 cells. After 24 hrs, the media was replaced with media containing only 2% FBS. At 48 hrs and 72 hrs after transfection, the media was harvested, centrifuged at 2000 rcf for 10 min, and the supernatant passed through a 0.45 um PVDF filter. The viral solution was then mixed with PEG6000 to a final concentration of 8% and 0.3M NaCl and incubated at 4°C for 12 hrs. Viral particles were collected by centrifugation at 2500 rcf for 45 min and resuspended in sterile PBS. Viral titers on the order of 10^10^ particles per ml were achieved.

**Quantitative Real-Time PCR**

Analysis of *Scn1a* knockdown was performed in B50 neuroblastoma cells (HPA Cultures #85042302), which were donated by David Schubert (Salk Institute, San Diego, CA) and grown according to HPA recommendations. Cells were plated at an approximate density of 40,000 cells/cm^2^ and were infected with the lentivirus (10 ul per 10 cm plate) after 1 day in culture. Cells were then harvested 4 days later. RNA was extracted using the RNeasy Plus Mini kit (Qiagen) following the manufacturer’s protocol. Cells were lysed directly in the plate by addition of homogenization buffer and homogenized with the QIAshredder spin column prior to RNA extraction. All RNA samples were tested for quality by spectrophotometric analysis at the Dartmouth genomics core facility and concentrations were determined. Equal amounts of RNA were reverse transcribed to cDNA using the High-capacity cDNA Reverse Transcription kit (Applied Biosystems, Life Technologies, Inc.) according to the manufacturer’s protocol. Equal volumes of cDNA were subsequently used for real-time quantitative PCR (RT-PCR) with primer sets for *Scn1a* (Applied Biosystems Assay ID# Rn00578439), *Scn2a* (Rn00561862) and *GAPDH* (Rn99999916). RT-PCR was performed using the Applied Biosystems Taqman Gene Expression assays with FAM dye-labeled, TaqMan MGB probes and the Applied Biosystems 7500 Real-time PCR machine following the manufacturer’s guidelines. Reactions were performed in triplicate for each sample and averaged. *Scn1a* and *Scn2a* expression levels are reported as normalized to *GAPDH*.

**Immunohistochemistry**

Immunofluorescence was performed for analysis of Na_v_1.1 expression in the MSDB. Rats were anesthetized with isoflurane and perfused intracardially with phosphate buffered saline (PBS) followed by 4% paraformaldehyde (PFA) in PBS. The brains were then rapidly removed and incubated in 4% PFA for 4 hrs at 4° C prior to cryoprotection in 30% sucrose. Brains were then frozen in Tissue Tek O.C.T. embedding compound (Andwin Scientific), cut at 35 µm and collected as free-floating sections in PBS. Sections were washed in PBS and blocked with 5% goat serum, 1% bovine serum albumin, and 1% Tween-20 in PBS for 1 hr at room temperature. Sections were then incubated with primary antibodies in blocking buffer for 36 hrs at 4° C: rabbit anti-Na_v_1.1 (1:50, Chemicon/Millipore), mouse anti-parvalbumin (1:1000, Chemicon/Millipore), chicken anti-GFP (1:1000, Abcam), mouse anti-GAD67 (1:200, Chemicon/Millipre), or goat anti-choline acetyl-tranferase (ChAT; 1:500, Chemicon/Millipore). For sections incubated with anti-ChAT antibody, goat serum was omitted from all buffers. Sections were subsequently washed in PBS for 30 min and incubated in AlexaFluor-conjugated secondary antibodies (1:750, Invitrogen) in blocking buffer for 3 hrs at room temperature. Negative controls included sections incubated with primary anti-Na_v_1.1 plus control antigen (Chemicon/Millipore) and sections incubated without primary antibody. Sections were counterstained and coverslipped with ProLong Gold mounting medium containing DAPI (Invitrogen, Life Technologies, Inc.).

Stained sections were imaged on an Olympus IX73 inverted fluorescent microscope (Olympus America, Inc.) and a Zeiss LSM 510 confocal microscope using a 2 µm optical slice thickness. Adjustments to brightness and contrast were made in Photoshop (Adobe, Inc.) for images used as examples. For quantification, immunofluorescence was performed on all sections together and images were acquired with a 40X objective under identical exposure settings. The mean fluorescent intensity for GFP-positive and GFP-negative Na_v_1.1-immunoreactive cells was calculated by tracing the cell circumference in ImageJ (NIH), and an area without Na_v_1.1 staining was selected for subtraction of background signal.

**Stereotaxic Injections**

Rats were anesthetized with isoflurane (1-3% in oxygen) during surgery. After the dorsal surface of the skull was exposed, two holes were drilled anterior to bregma at the following coordinates [3]: AP +0.7, ML +/- 1.4 (units in mm). A total of 2 µl of the viral solution was infused into the MSDB from each side using a 10 µl Hamilton syringe with a 30 gauge needle (Hamilton Company USA). The syringe was first lowered at an angle of 13 degrees (from vertical) into the MSDB to the following target coordinates: AP +0.7, ML 0.0, DV 6.6. The syringe was then lowered further by 200 µm and 0.5 µl of the viral solution was infused at a flow rate of 2.5 ul/min. The syringe was then raised by 200 µm increments three more times, and each time an additional 0.5 µl was infused. The syringe was left in place for 3 min after each infusion. This was done with the syringe lowered into the MSDB from both the left and right sides. An example of the injection site is shown in Fig 2C. Further experimental procedures were not performed until at least two weeks following injections.

**Single-Unit and LFP Recordings under Urethane**

Between 2-6 weeks after MSDB injections, each rat was anesthetized by intraperitoneal (i.p.) injection of 1.5 g/kg Urethane (Sigma-Aldrich Co.) and placed in a stereotaxic frame. The dorsal surface of the skull was exposed and small holes were drilled for the locations of each electrode. A small rectangular piece of skull located over the MSDB was then removed, and the superior sagittal sinus was ligated on two ends and cut to allow direct access of the recording electrode to the MSDB along the midline. A ground wire was inserted below the surface of the skull at approximately AP -8.0, ML 5.0(L) and fixed to the skull surface by Super Glue. A custom electrode was designed for bipolar LFP recordings with the tips of the wires were cut at an angle so that they spanned ~1 mm between the longest and shortest. This electrode was stereotaxically lowered into the dorsal hippocampus, spanning the CA1 region, at the following coordinates measured from the longest tip of the electrode: AP -4.0, ML 3.0(R), DV 2.8. A 16-channel silicon probe (NeuroNexus Technologies) used for single-unit recordings was then stereotaxically lowered to the dorsal tip of the MSDB at the following coordinates: AP +0.7, ML 0.0, DV 5.6. This probe was designed with 4 tetrodes, 2 per shank spaced 150 µm apart, and sensor sites of 20 µm diameter. Hippocampal electrode signal was referenced to an electrode placed in the overlying corpus callosum and septum electrodes were referenced to the animal ground. The ground wire, LFP electrode, and single-unit probe were each connected to the EIB-27 Nexus 16 Adapter (Neuralynx, Inc.), and the signal was pre-amplified via the HS-27 headstage amplifier (Neuralynx, Inc.) and sent to a Neuralynx recording system equipped with Cheetah32 acquisition software (Neuralynx, Inc.). All electrodes were coated with DiI (1,1'-Dioctadecyl-3,3,3',3'-Tetramethylindocarbocyanine Perchlorate; Molecular Probes, Life Technologies, Inc.) prior to implantation to facilitate visualization of the electrode tracks after each experiment.

MSDB single-units and hippocampal LFPs were simultaneously recorded during 10 minute sessions, with the depth of the single-unit probe being lowered each time by 200 µm in order to span the vertical length of the MSDB. If no units were detected, the single-unit probe was lowered further by 200 µm. Single-units were filtered at 600-6000 Hz, sampled at 30 kHz and an amplitude threshold was set at minimum of 50 µV. LFP data were filtered at 0.1-9000 Hz and sampled at 10 kHz. During each recording session, spontaneous theta oscillations were typically observed on the hippocampal LFP. In addition, theta oscillations were induced by pinching the tail of the rat, on average 3 times throughout the session spaced equally apart in time by approximately 3-4 min (also see Fig S3C,E). When the single-unit probe reached the ventral portion of the MSDB (at which point units were rarely detected), the electrodes were raised, the animal was perfused intracardially with 4% PFA as described above, and the brain was preserved for histology. Injection sites were confirmed by visualization of GFP expression, and all electrode tracks were confirmed by visualization of DiI (also see Fig 3B and S3B Fig). If the injection site was not located within the MSDB or the electrode track did not pass through the injection site, the recording was excluded from the analysis.

**Open Field and Morris Water Maze**

Rats that had been injected with the lentivirus 2-6 weeks prior were tested on the Open Field and Morris Water Maze. The Open Field consisted of a circular arena (a grey cylinder of 76 cm diameter and 40 cm height) with a white cue card on the inner wall spanning a 100° arc of the cylinder. The arena was located within a separate room illuminated by ceiling lights. Prior to testing, all rats were habituated to the experimenter by handling, but the rats were not pre-exposed to the testing arena. On the day of testing, each rat was placed in the center of the arena and allowed to voluntarily explore for 10 min. The arena floor was cleaned with water in between rats. Sessions were recorded by an overhead video camera connected to a DVD recorder, and the rat’s position was extracted by contrast detection using ANY-maze software (Stoelting, Inc.).

The Water Maze procedure was similar to previous studies in our lab [4]. The arena consisted of a circular stainless-steel pool (2 m diameter, 50 cm high), located in a dedicated behavior room with two well-lit distal room cues spaced approximately 135 degrees apart that remained constant for the entire experiment. The pool was partially filled with room temperature water and mixed with non-toxic white acrylic paint. The pool was divided into four equal quadrants and a 10 cm diameter platform was placed in the center of one quadrant, submerged approximately 2 cm below the surface and 35 cm from the wall. On the first day, each rat was placed into the pool without the platform and allowed to swim freely for 2 min. This Habituation session allowed the rat to become familiar with the environment and allowed the experimenter to observe the baseline exploratory behavior. For the next 4 consecutive days, 4 trials per day were conducted in which each rat was placed into the pool from one of four entry points and allowed 2 min to find and climb onto the hidden platform. After climbing onto the platform, the rat was allowed to remain there for 30 s before proceeding to the next trial. If a rat did not find the platform in 2 min, it was guided there by the experimenter and allowed to remain on the platform for 30 s. The platform location remained fixed, but the sequence of the entry points was randomized each day. On the last day, a Probe trial was conducted approximately 4 hrs after the last trial, in which each rat was placed in the pool without the platform and allowed to swim for 2 min. Typically, rats that remember the platform location will spend more time searching in the quadrant where the platform was previously located. Following the Probe trial, each rat was given 4 re-training trials during which the platform was returned to its previous location. Over the next two days, a Reversal was conducted, in which the platform location was changed (to the opposite quadrant on the first day, and an adjacent quadrant on the second day). Each day, the rat was given 4 trials as before. On Reversal trials, rats typically learn the new platform location faster because they are already familiar with the environment. All sessions were recorded by an overhead video camera connected to a DVD recorder, and the rat’s position was extracted by contrast detection using ANY-maze software (Stoelting, Inc.). The latency to finding the platform was the primary outcome measure, but path efficiency (path distance / straight-line distance from entry to platform) was also assessed.

**T-maze Rewarded Alternation & Hippocampal LFP Recordings**

Rats that had been previously injected with the lentivirus containing either the control vector or sh*Scn1a*-2 (sh-2) were subsequently implanted with hippocampal LFP recording electrodes and were tested on a rewarded alternation task in the T-maze. A custom LFP recording electrode similar to that used for urethane recordings (described above) was surgically implanted under isoflurane anesthesia into the dorsal hippocampus CA1 region at the following coordinates (measured from the tip of the longest wire): AP -4.0, ML 3.0, DV 2.8 (mm from Bregma). Three small screws were attached to the skull surface and the implants were fixed to the skull and screws with dental cement. All animals were allowed two weeks to recover before behavioral procedures were performed.

The T-maze apparatus (also see Fig 6A) consisted of a central arm 100 cm in length and two end arms spanning 135 cm. Sliding doors were located at the junction of each end arm and 20 cm from the beginning of the central arm (the start box). The width of the arms was 15 cm and the height of the walls was 30 cm. At the end of each arm was a food cup. The T-maze was painted black and placed in a room with dim overhead lighting and two well-lit distal cues against the room walls offset from the end arms of the T-maze. Prior to testing, rats were food-restricted to 85% of their starting body weight. Rats were first habituated to the T-maze and the food reward (Cocoa Puffs, General Mills, Inc.), and gradually trained to run from the start box to the end arm to receive the reward. Habituation and pre-training lasted one week and followed guidelines described previously [5]. Only rats that ran from the start to the food reward for 12 consecutive trials were included in subsequent testing.

Rats were then tested on a non-match-to-place protocol for 6 days with 12 trials per day. Each trial consisted of a Sample run followed by a Choice run. On the Sample run, the rat was placed in the start box and allowed to run to only one of the end arms to receive the reward. In this Sample run, the door was closed blocking the opposite arm. Once the rat consumed the reward, it was removed from the maze, both doors were then opened, and a new reward was placed in the opposite arm. In the next Choice run, the rat was then placed back in the start box and again allowed to run to the end, at which point it had to choose the arm not previously visited to successfully receive the reward. The rats rarely stopped as they entered one arm, running all the way to the end to obtain the reward. An entry was counted as soon as the rat placed all four paws into one arm. On the Sample run, a left versus right turn was selected according to a pseudorandom sequence with the criteria that each arm be selected an equal number of times each day and for no more than 3 trials in a row [6]. All rats also received the same sequence of left/right turns on a given day. During the first 2 days of testing, there was no delay interval incorporated between the Sample and Choice runs, with the exception of the time it took to return the rat to the start box (~10s). However, on days 3 to 6, a variable delay was incorporated between the Sample and Choice runs. Once the rat was returned to the start box after the Sample run was completed, a delay of either 15, 30 (‘short’) or 60 (‘long’) seconds was enforced before the rat was allowed to run for the reward in the Choice run [7]. The delay length was also randomized between trials with the criteria that each delay occurred once within each block of 3 trials, and all rats received the same sequence of delays on a given day. Each rat performed 12 trials consecutively before testing for the next rat was started. To minimize use of odor cues, a large cup of the food reward was placed outside of the maze at each end arm (out of sight to the rat) during all trials. Thus, food odor did not indicate the correct location of the reward in the end arms.

During behavioral testing, the rat’s position was tracked by two LEDs that were attached to the rat headstage and recorded via a CCD video camera connected to a frame grabber integrated within the Neuralynx recording system. Signal from the electrodes was preamplified directly on the rat’s headstage by operational amplifiers mounted as followers (gain = 1) and sent to a Neuralynx recording system (Neuralynx Inc.). Hippocampal LFPs were recorded as described above for urethane recordings.

**Data Analysis**

Single-unit discrimination was performed offline with SpikeSort 3D software (Neuralynx, Inc.) using the peak and valley amplitudes of the waveform on each of the four channels of a tetrode. When units could not be distinguished by these features alone, principal component analysis was also used. Spike-time autocorrelations and inter-spike-interval histograms were visually inspected for all units. Units without a clear refractory period or with less than 100 spikes in a session were excluded. All additional analyses for single-units and LFP data were performed using custom-written programs in Matlab (Mathworks, Inc.) with use of the Chronux toolbox ([www.chronux.org](http://www.chronux.org)).

Considering the role of voltage-gated sodium channels in action potential generation and propagation, the mean and peak firing frequencies were the primary measures investigated for single-unit data. For each unit, timestamps for spike data were extracted and a histogram of inter-spike-intervals (ISI) was constructed. The peak firing frequency was defined as the frequency (the inverse of the ISI) corresponding to the first local maxima on the ISI histogram. Action potential width was also assessed, calculated as the peak-to-trough time of the average waveform. In addition, the proportion of units that exhibited fast-firing properties (including tonic- and burst-firing) were quantified by setting thresholds based on the peak and mean firing frequency. At a basic level, units with a peak and mean firing frequency at an approximate 1:1 ratio exhibit a tonic firing pattern, while units with a peak firing frequency much greater than the mean exhibit burst-firing properties. A scatter plot of these firing parameters for all units helped to identify units of each type (S2B Fig). The ratio of peak/mean firing frequency was computed for all units, and they were categorized based on the following criteria: 1) fast-firing, tonic units had a peak/mean ratio of less than 2 and a mean frequency greater than 30 Hz and corresponded with units in area #1 in S2B Fig; fast-firing, bursty units had a peak/mean ratio greater than 2 and a peak frequency greater than 75 Hz and corresponded to area #2 in S2B Fig.

For LFP data recorded under urethane anesthesia, time-frequency power spectra were computed using the multi-taper method (window size 4s, step 1s). To compare between rats, power values were normalized by dividing by the sum of the power spectrum from 1 to 120 Hz. It should be noted that under urethane anesthesia the frequencies of hippocampal oscillations are slower and were therefore designated as follows. Theta power was defined as the sum of the normalized power between 2 to 5 Hz, as indicated by a prominent peak in this range (peak near 3.5 Hz) in the individual spectrograms (also see S3C Fig) and as has been noted in prior studies [8,9]. Theta frequency was defined as the frequency corresponding to the local maxima within the theta band, and gamma power was defined as the sum power between 25 to 50 Hz.

Theta epochs were also analyzed separately using two methods. In the first method, 30s of LFP data were extracted time-locked to the tail pinch event (also see Fig 4H). However, induction of theta after the tail pinch was variable in the sh*Scn1a* groups. Therefore, a second method was also employed using an automatic theta detection algorithm that was created to detect only periods when hippocampal theta rhythm was present. Times when the normalized theta power (defined above) exceeded an empirically-determined threshold of 0.94 a.u. were extracted (S3F Fig). These timestamps were then used to calculate the percent time spent in theta, as well as the mean theta frequency, theta power and gamma power during theta epochs. The robustness of these results based on a given theta threshold was evaluated by systematically varying the threshold and re-computing these values (S3G Fig).

Timestamps for automatically-detected theta epochs were subsequently used to investigate the phase-locking of MSDB units to hippocampal theta rhythm. The LFP signal was first band-pass filtered for theta frequencies (2-5 Hz), and theta phase was determined by the Hilbert transform. For each unit, spike timestamps were associated with the corresponding theta phase by interpolation. Spikes that occurred outside of theta epochs were then discarded. The mean resultant vector length was calculated from the theta phase of the spikes (circular statistics toolbox, Matlab), and units with significant theta phase-locking were identified using the Rayleigh test for non-uniformity with a significance level of α=0.05 [10]. The mean vector length was considered as an indicator of the strength of theta phase-locking.

For LFP data recorded during performance in the T-maze, time-frequency power spectra were computed using the short-time Fourier transform (window size 0.5s, step 0.1s). Power values were normalized as described above by dividing by the sum of the power spectrum from 1 to 120 Hz. Theta power was defined as the sum of the normalized power between 5 to 12 Hz, and theta frequency was defined as the frequency corresponding to the local maxima within the theta band. Slow gamma power was defined as the sum power between 30 to 50 Hz and fast gamma between 65 to 120 Hz [11]. Theta power and frequency were first evaluated as a function of binned running speed. To further investigate the dynamic changes in theta rhythm as the rat navigated the T-maze, the maze was subsequently divided into 10 position bins (also see Fig 7D) and theta values were determined for each bin. For this analysis, only periods of movement were included by applying a speed threshold of greater than 5 cm/s.

Statistics. Unless indicated otherwise, a student’s t-test was used to compare differences between the control and shScn1a groups, and individual comparisons were tested between controls, shScn1a-1 (sh-1) and shScn1a-2 (sh-2) groups separately using an ANOVA followed by a Bonferroni test for multiple comparisons. To compare the distributions of single-unit firing frequency (Fig 3F,G) and theta phase-locking strength (Fig 5D) between groups, a two-sample Kolmogorov-Smirnov test (KStest) was used. To compare proportions between groups, a two-sample chi-square test was used (Figs 3H, 5E & S4C Fig). In the T-maze analysis, a Pearson correlation for theta frequency versus performance (choice accuracy) was performed and a linear regression was used to estimate the best fit line (Fig 7H).

**References**

1. Bender AC, Natola H, Ndong C, Holmes GL, Scott RC, Lenck-Santini P-P. Focal Scn1a knockdown induces cognitive impairment without seizures. Neurobiol Dis. 2013;54: 297-307.

2. Luikart BW, Schnell E, Washburn EK, Bensen AL, Tovar KR, Westbrook GL. Pten knockdown in vivo increases excitatory drive onto dentate granule cells. J Neurosci. 2011;31: 4345–54.

3. Paxinos G, Watson C. The Rat Brain in Stereotaxic Coordinates Fourth Edition. Academic press. San Diego, CA; London, UK: Academic Press; 1998.

4. Lenck-Santini P-P, Holmes GL. Altered phase precession and compression of temporal sequences by place cells in epileptic rats. J Neurosci. 2008;28: 5053–62.

5. Deacon RMJ, Rawlins JNP. T-maze alternation in the rodent. Nat Protoc. 2006;1: 7–12.

6. Fellows BJ. Chance stimulus sequences for discrimination tasks. Psychol Bull. 1967;67: 87–92.

7. Kelsey JE, Vargas H. Medial septal lesions disrupt spatial, but not nonspatial, working memory in rats. Behav Neurosci. 1993;107: 565–74.

8. Bland BH, Oddie SD, Colom LV. Mechanisms of neural synchrony in the septohippocampal pathways underlying hippocampal theta generation. J Neurosci. 1999;19: 3223–37.

9. Varga V, Hangya B, Kránitz K, Ludányi A, Zemankovics R, Katona I, et al. The presence of pacemaker HCN channels identifies theta rhythmic GABAergic neurons in the medial septum. J Physiol. 2008;586: 3893–915.

10. Fisher NI. Statistical Analysis of Circular Data. Cambridge University Press. 1993.

11. Colgin LL, Denninger T, Fyhn M, Hafting T, Bonnevie T, et al. Frequency of gamma oscillations routes flow of information in the hippocampus. Nature. 2009;462: 353-7.
